# Clinical and transcriptomic characterization of patients with chronic lymphocytic leukemia harboring t(14;19): an ERIC study

**DOI:** 10.1038/s41375-025-02755-8

**Published:** 2025-09-19

**Authors:** Andrea Visentin, Enrico Gaffo, Moritz Fürstenau, Kerry A. Rogers, Baliakas Panagiotis, Chenghua Cui, Cecelia Miller, Claudia Haferlach, Karla Plevova, David Oscier, Zadie Davis, Florence Nguyen-Khac, Eleonora Roncaglia, Gian Matteo Rigolin, Anastasia Athanasiadou, Fanny Baran-Marszak, Alberto Valiente, Maria José Terol, Pau Abrisqueta, Blanca Espinet, Anna Puiggros, Annalisa Martines, Laura Bonaldi, Francesca Romana Mauro, Lydia Scarfò, Thomas Chatzikonstantinou, Eugen Tausch, Karl-Anton Kreuzer, Arnon P Kater, Francesc Bosch, Michael Doubek, Panagiotis Panagiotidis, Olga Kalashnikova, Federica Frezzato, Giulia Calabretto, Valeria Ruocco, Silvia Orsi, Alessandro Cellini, Francesco Angotzi, Andrea Serafin, Shuhua Yi, Barbara Eichhorst, Jennifer A. Woyach, Antonio Cuneo, Paolo Ghia, Kostas Stamatopoulos, Livio Trentin, Stefania Bortoluzzi

**Affiliations:** 1 Hematology Unit, Department of Medicine, University of Padua, Padova, Italy; 2 Department of Molecular Medicine, University of Padua, Padova, Italy; 3 Department I of Internal Medicine, University of Cologne, Cologne, Germany; 4 The Ohio State University Comprehensive Cancer Center, Columbus, OH USA; 5 Department of Immunology, Genetics and Pathology & Clinical Genomics Uppsala, Science for Life Laboratory, Uppsala University, Uppsala, Sweden; 6 State Key Laboratory of Experimental Hematology, National Clinical Research Center for Blood Diseases, Haihe Laboratory of Cell Ecosystem, Institute of Hematology & Blood Diseases Hospital, Chinese Academy of Medical Sciences & Peking Union Medical College; Tianjin Institutes of Health Science, Tianjin, China; 7 MLL Munich Leukemia Laboratory, Munich, Germany; 8 Department of Internal Medicine, Hematology and Oncology, University Hospital Brno & Faculty of Medicine, Masaryk University, Brno, Czech Republic; 9 Molecular Pathology, University Hospitals Dorset, Royal Bournemouth Hospital, Bournemouth, United Kingdom; 10 Hôpital Pitié-Salpêtrière, Service d’Hématologie Biologique, Paris, France; 11 Computational Genomics Group, Department of Oncology, Surgery and Gastroenterology, University of Padua, Padova, Italy; 12 Department of Biology, University of Padua, Padova, Italy; 13 Hematology Section, Department of Medical Sciences, Azienda Ospedaliera-Universitaria, Arcispedale S. Anna, University of Ferrara, Ferrara, Italy; 14 Hematology Department and HCT Unit, G. Papanicolaou Hospital, Thessaloniki, Greece; 15 Laboratoire d’hématologie, Hopital Avicenne, Assistance Publique-Hôpitaux de Paris, Paris, France; 16 Servicio de Genética. Hospital Universitario de Navarra, Pamplona, Spain; 17 Servicio de Hematología y Oncología Médica, Hospital Clinico Universitario de Valencia, Valencia, Spain; 18 Department of Hematology, Vall d’Hebron Institute of Oncology (VHIO), Hospital Universitari Vall d’Hebron, Barcelona, Spain; 19 Molecular Cytogenetics Laboratory, Pathology Department, Hospital del Mar; Translational Research on Hematological Neoplasms Group, Cancer Research Program, Hospital del Mar Research Institute, Barcelona, Spain; 20 Immunology and Molecular Oncology Unit, Veneto Institute of Oncology IOV-IRCSS, Padova, Italy; 21 Department of Translational and Precision Medicine, Hematology unit, ‘Sapienza’ University, Rome, Italy; 22 Medical School, Università Vita-Salute San Raffaele, Milano, Italy; 23 Division of Experimental Oncology, IRCCS Ospedale San Raffaele, Milano, Italy; 24 Institute of Applied Biosciences, Center for Research and Technology Hellas, Thessaloniki, Greece; 25 Ulm University, Ulm, Germany; 26 Department of Hematology, Cancer Center Amsterdam and Lymphoma and Myeloma Center Amsterdam, The Netherlands, Amsterdam, Netherlands; 27 First Department of Propaedeutic, University of Athens, Athens, Greece; 28 Federal State Budgetary Educational Institution of Higher Education Academician I.P. Pavlov First St. Petersburg State Medical University of the Ministry of Healthcare of Russian Federation, St. Petersburg, Russian Federation

**Keywords:** Translational research, Cancer genomics

## Abstract

In chronic lymphocytic leukemia (CLL), the role of complex karyotype (CK) for prognostic stratification remains a topic of debate, and the impact of specific cytogenetic abnormalities is still unclear. This study aims to investigate the clinical and biological features of CLL with t(14;19)(q32;q13) (tCLL) involving the BCL3 gene. Patients with tCLL were younger and more commonly presented unmutated IGHV gene, subset #8 stereotypy, trisomy of chromosome 12, and complex karyotype than other patients without t(14;19) (oCLL). The presence of t(14;19) was associated with a shorter time to treatment and overall survival compared to oCLL. Gene expression analysis revealed a unique transcriptome profile in tCLL, characterized by the upregulation of BCL3 and the activation of B-cell receptor, PI3K-Akt. Conversely, apoptosis-related pathways were suppressed in tCLL. While the BTK gene was upregulated, the BCL2L11 gene, coding for the pro-apoptotic protein BIM, was downregulated. Notably, patients with tCLL were characterized by a trend (*p* = 0.058) for a longer time to the next treatment with BTK inhibitors (BTKi) compared to those treated with a venetoclax-based (Ven-based) regimen. We underscore the adverse outcomes of tCLL, its distinct molecular features and gene expression patterns. Therefore, our data suggest that identifying tCLL could help tailor therapeutic approaches.

**Figure:**
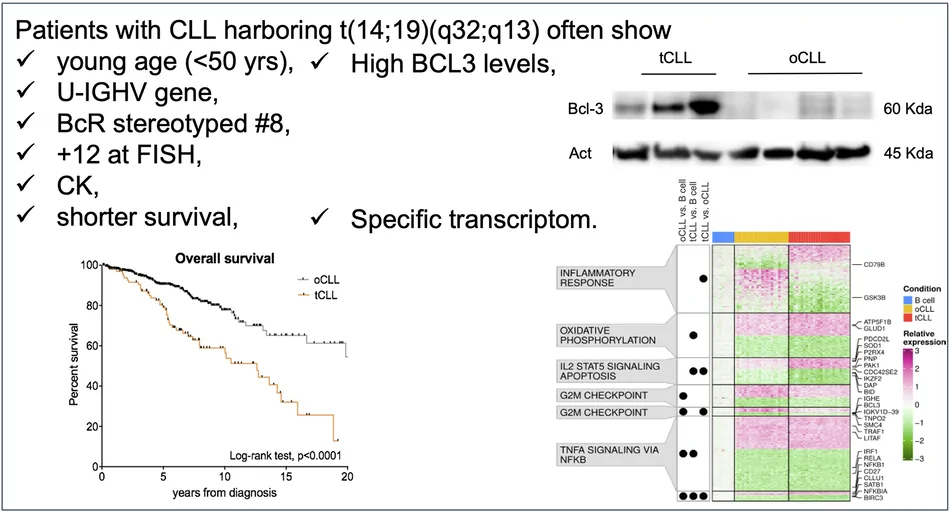

## Background

Stimulated chromosome banding analysis (CBA) has recently been included in the biological criteria for stratification of patients with chronic lymphocytic leukemia (CLL) in some guidelines [1, 2] together with immunoglobulin heavy variable (IGHV) gene mutational analysis and *TP53* abnormalities, i.e., 17p13 deletion assessed by interphase fluorescence in situ hybridization (FISH) and *TP53* mutation. CBA can identify chromosome abnormalities that would have been missed by FISH [3–6]. A complex karyotype (CK), defined as the presence of at least 3 chromosomal abnormalities, can be identified in almost 20% of patients at the time of CLL diagnosis and up to 40% among relapsed/refractory cases [3, 7–9]. CK has been associated with *TP53* abnormalities, chemo-refractoriness, early relapse, and a higher risk of Richter transformation [3–7]. At variance with other hematological disorders, a prognostic value has been associated only with the presence of at least 5 chromosomal abnormalities in the absence of *TP53* aberrations (deletion of 17p and/or *TP53* mutations). These subgroups of patients, generally defined as high-CK, had the worst outcome, even if managed with targeted therapies [4, 7, 8, 10–12]. Despite the reported data above, the role of CK remains a subject of debate.

Previous studies have reported that t(14;19)(q32;q13), which causes the relocalization of the B-cell leukemia/lymphoma 3 (*BCL3*) gene near regulatory elements of the immunoglobulin heavy chain (IGH), leads to *BCL3* overexpression [13–15]. It is a very rare cytogenetic abnormality found in <1% of CLL and other B-cell malignancies. Some studies have indicated that CLLs with t(14;19) (tCLL) have an adverse prognosis [13–20]; however, these findings were not confirmed by others [21].

The function of BCL3 is not entirely known [22, 23]. BCL3 is an atypical member of the regulatory IκB family, which, unlike classical members, is a nuclear transcription cofactor. BCL3 can bind to NF-κB p50 and p52 homodimers, repressing or activating a subset of NF-κB-regulated genes. It can also inhibit the ubiquitination and subsequent proteasomal degradation of p50 homodimers, thereby stabilizing their DNA binding. However, it can also recruit a corepressor, inhibiting the transcription [22–25].

In this study, we aimed to define the clinical and biological features of tCLL, utilizing a large cohort of 101 patients from the European Research Initiative on CLL (ERIC) network, including 66 patients who were also treated with targeted agents. Furthermore, by sequencing the transcriptome of a sizable patient cohort, we aimed to characterize the expression profile of tCLL and gain a deeper understanding of the biological basis of this CLL subtype.

## Materials and methods

### Study design

The inclusion criteria for this study were a diagnosis of CLL according to the 2018 International Workshop on Chronic Lymphocytic Leukemia (iwCLL) criteria, age >18 years, CBA performed within one year from diagnosis, presence of t(14;19)(q32;q13) and Matutes score of 3 or above. Patients with a Matutes score of 3 were defined as atypical CLL. The data included in the comparative analysis were gender, age, Binet stage, need for treatment, cytogenetics detected by fluorescence in situ hybridization (FISH), IGHV gene mutational status, stereotyped B cell receptor (BCR), and *TP53* abnormalities, including deletions of chromosome 17p and/or *TP53* mutations [26]. Patients were treated according to the iwCLL guidelines. The primary endpoints were the time to first treatment (TTFT) and overall survival (OS) in patients with the t(14;19) translocation. The secondary endpoints included the time to the next treatment (TTNT) and a comparison of clinical-biological variables between tCLL and 540 karyotyped CLL patients without t(14;19) (oCLL) from the University of Padova. Patients were treated with fludarabine-cyclophosphamide-rituximab (FCR) / bendamustine-rituximab (BR), continuous BTK inhibitors (BTKi), and a time-limited venetoclax-based therapy (Ven-based). This study was approved by the local research ethics committee (4430/AO/18), and informed consent was obtained from all patients. Statistical analyses were done as reported in the Supplementary Materials.

### Biological markers

IGHV gene mutational status, interphase FISH, and *TP53* mutations were assessed in each of the collaborating centers following current ERIC recommendations as described in the Supplementary Materials.

### RNA-seq sequencing

For RNA sequencing (RNA-seq), RNA was extracted from 10^6^ leukemic B cells (RIN > 7) from 25 CLL patients with t(14;19), plus 22 CLL patients with normal karyotype/FISH or trisomy 12 ( + 12) and from mature B cells from 5 age-matched healthy individuals and sequenced by Illumina Novaseq (150 bp per end, 120 × 10^6^ reads/sample). Four additional mature B-cell samples from healthy donor RNA-seq datasets were obtained from a previous study [27]. The expression of the BCL3 protein was assessed by Western blot analysis, as described in the Supplementary Methods. The expression of key selected genes or protein levels was validated by RT-qPCR or flow cytometry, as described in the supplementary methods. Primer sequences are listed in Table S1.

### Bioinformatics analysis

RNA-seq data was analyzed with CirCompara2 v0.1.2.1 [28–32] using the GRCh38 human genome reference and ENSEMBL gene annotation v108. The differential expression was assessed using DESeq2 v1.40.1 [33], including removing batch effects using the sva algorithm v3.48.0 [34] and considering significant differences with Benjamini-Hochberg adjusted *p* values (p-adj) ≤ 0.01.

Gene set and pathway enrichment analysis were performed through the clusterProfiler v4.8.1 Bioconductor package. All analyses were performed with custom scripts in an R v4.3.0 environment. Results of gene set enrichment analysis [35–38] were considered significant for p-adj ≤ 0.05. Topology-based analyses were performed using the SPIA [39] package. The statistical significance of BCL3 target gene enrichment among overexpressed genes in tCLL was assessed using a hypergeometric test performed with R’s *phyper* function, utilizing parameters derived from the TFLink database and our gene expression data [40].

## Results

### Patient characteristics

We enrolled 101 CLL patients with t(14;19) (tCLL) from 23 centers (Table 1; Supplementary Fig. S1): 59% were male, and the median age was 57 ± 12 years. Of note, at CLL diagnosis, 30% of the patients were younger than 50 years, 59% were at Binet stage A, 28% at stage B, and 13% at stage C. IGHV mutational status was available for 85 patients, of which 91% carried unmutated IGHV genes (31% presented IGHV4-39). Patients were diagnosed between 1994 and 2022. The Sanger sequencing of IGHV analysis was available for 38 patients, and the stereotyped BCR subset #8 was found in 12 (31.6%) cases. FISH data were available from 97 patients: 5% harbored 13q-, 8% 11q-, 57% +12, 9% 17p, and 20% none of these abnormalities detected. *TP53* abnormalities were detected in 14% of the patients (9 deletions and 5 mutations). Regarding karyotype, 52% displayed a CK3 (11 with and 36 without TP53 abnormalities, with 6 missing data), and 20% a CK5 (8 with and 10 without TP53 abnormalities, with 2 missing data). None had multiple trisomies. It is worth noting that the features of patients with tCLL were skewed compared to those with oCLL (Supplementary Table S2). Patients with tCLL were younger and more commonly showed unmutated IGHV genes, as well as +12 and BCR subset #8 (Supplementary Tables S2, S3).

**Table 1:** Clinical and biological features of patients.

	Patients
**Age (years)**	
Median ± sd	57 ± 12
**Gender**	
Female	41 (41%)
Male	60 (59%)
**Binet stage**	
A	60 (59%)
B	28 (28%)
C	13 (13%)
IGHV status*	
M-IGHV	10 (12%)
U-IGHV	75 (88%)
**BCR stereotyped°**	
Subset #8	12 (32%)
**FISH**^**+**^	
13q-	5 (5%)
“Normal”	19 (20%)
+12	55 (57%)
11q-	8 (8%)
17p-	9 (9%)
***TP53*** **abn**^**+**^	
Normal	83 (86%)
Disrupted	13 (14%)
**Karyotype**	
No CK	48 (48%)
CK3	53 (52%)
CK5	20 (20%)
**Richter transformation**	
Cases	13 (13%)

*sd* standard deviation, *M-IGHV* mutated IGHV gene, *U-IGHV* unmutated IGHV gene, *13q-* 13q14 but interphase FISH analysis, *11q-* del11q22-23 by interphase FISH analysis, *17p-* 17p13 by interphase FISH analysis. *TP53 abn* TP53 abnormalities include deletions and/or mutations, *CK3* complex karyotype, ⩾3 chromosome abnormalities, and CK5 = ⩾5 chromosome abnormalities, *n.a.* not available.

* Data available from 85 patients. °Data available from 38 patients. + Data available from 96 patients.

### The t(14;19) translocation has a negative prognostic impact

After a median follow-up of 6.92 years, 92% of the patients needed at least one treatment (median 2, range 1–11), 13% developed Richter transformation, and 43% died (23% infections, 12% CLL progression, 19% Richter transformation, 12% other causes, 26% unknown).

**Fig. 1:**
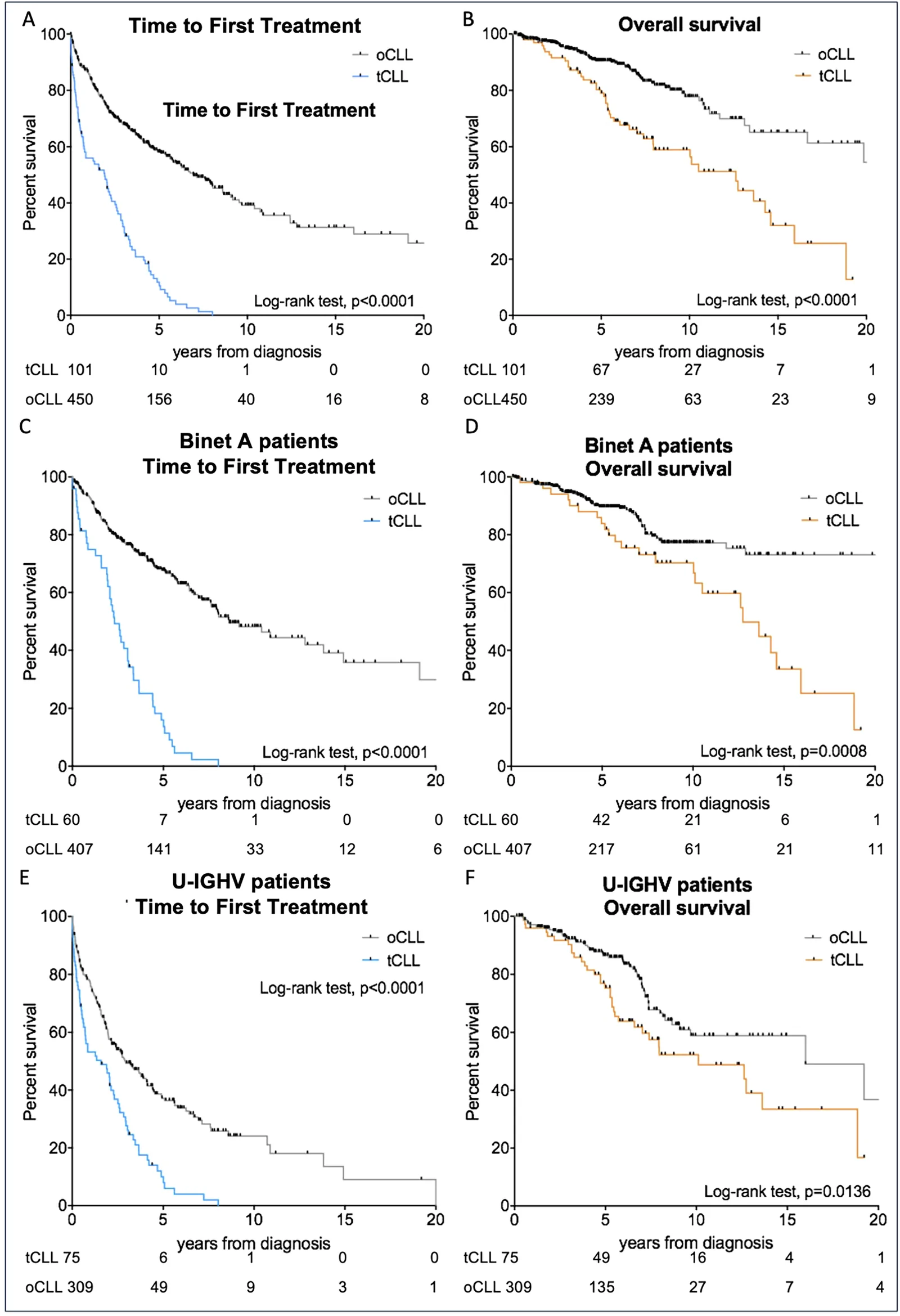
Kaplan-Meier curves for survival analysis. The Kaplan-Meier curves of (**A**) time to first treatment and **B** overall survival of patients with t(14;19) (*n* = 101, tCLL) and other karyotyped CLL without t(14;19) (*n* = 450, oCLL), in patients at Binet A stage at diagnosis (**C**, **D**) and harboring unmutated IGHV genes (U-IGHV, **E**, **F**).

The time to first treatment (TTFT) in tCLL patients was shorter than in oCLL patients, with a median TTFT of 1.92 years and 6.94 years, respectively (*p* < 0.0001). After 7 years of follow-up, 97% and 50% of patients with and without t(14;19) started a second line of treatment, respectively (HR 3.55, 95% CI [2.46–5.12], Fig. 1A). The median overall survival (OS) was 12.6 years in tCLL, whereas it was not reached in oCLL (*p* < 0.0001). The 7-year OS was 66% and 97% for tCLL and oCLL, respectively (HR 2.53, 95% CI [1.59–4.03], Fig. 1B). The same differences were confirmed also when focusing only on patients at Binet A stage (60 tCLL and 407 oCLL) and with unmutated IGHV gene (75 tCLL and 309 oCLL), with the presence of t(14;19) being associated with a worse outcome (TTFT, HR 4.27, 95% CI [2.44–7.46], *p* < 0.0001; OS 2.21, HR 95% CI [1.22–4.01], *p* = 0.0008; Fig. 1C, D; TTFT, HR 2.01, 95% CI [1.48–3.00], *p* < 0.0001; OS 1.70, HR 95% CI [1.06–2.71], *p* = 0.0136; Fig. 1E, F).

In univariate analysis, none of the variables assessed (age >65 years, male gender, U-IGHV, BcR subset 8, +12, *TP53* abn, CK, CK5) predicted a shorter TTFT or OS in patients with t(14;19) (Supplementary Table 9).

To better understand the negative prognostic value associated with t(14;19), we compared the TTFT and OS with those of oCLL patients who had well-known negative prognostic markers such as *TP53* abnormalities, CK, and CK5 [5]. Patients with t(14;19) and CK or +12 had a worse outcome compared to patients with CK or +12 without t(14;19); instead, their outcomes are superimposable to those of patients with *TP53* abnormalities or CK5, despite being infrequent in patients with tCLL (Supplementary Figure S2).

### The predictive value of t(14;19)

**Fig. 2:**
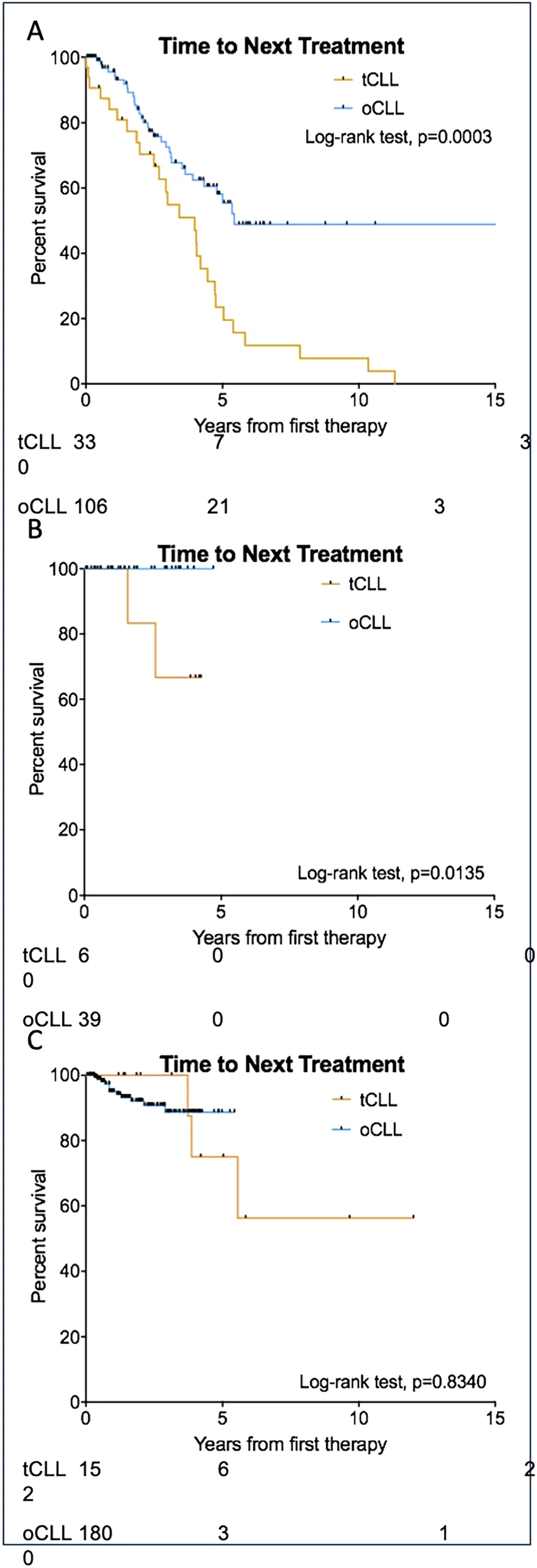
Kaplan-Meier of time to next treatment. The Kaplan-Meier curves of time to the next treatment of patients treated with t(14;19) (tCLL) and other CLL without t(14;19) (oCLL) who received (**A**) fludarabine-cyclophosphamide-rituximab (FCR) / bendamustine-rituximab (BR) (*n* = 32 and *n* = 107, respectively), **B** venetoclax-based therapy (*n* = 6 and *n* = 39) and **C** BTK inhibitor (*n* = 14 and *n* = 180) as frontline therapy.

We also assessed whether t(14;19) influenced the response to treatment and compared the TTNT after different therapies of tCLL and oCLL. Among the 93 tCLLs requiring treatment, 33 (36%) received chemoimmunotherapy with FCR or BR as first-line therapy, 15 (16%) a BTKi (11 ibrutinib, 2 acalabrutinib, 2 zanubrutinib), 6 Ven-based therapy, and 39 other therapies, such as single-agent chemotherapy or monoclonal antibody. After frontline therapy with FCR/BR, the median TTNT was 3.98 and 5.48 for tCLL and oCLL (*p* = 0.0003, HR 2.47, 95% CI [1.38–4.44]; Fig. 2A). After 3 years, 46% of tCLLs started a second line of therapy. Instead, only 28% of oCLLs required further treatment (*p* < 0.001, Chi-square and *p* = 0.0003, Log-rank test). In addition, as shown in Fig. 2B, after 3 years from the start of Ven-based therapy, 33% of tCLLs needed further treatment as opposed to no need for treatment of oCLLs (*p* = 0.0235, HR 57, 95% CI [2.3–134]). However, the TTNT was not statistically different for patients managed with BTKi as the first-line therapy (*p* = 0.8340, HR 2.47, 95% CI [1.37–4.44]). At 3 years, none of the patients with tCLL and 88% of those with oCLL treated with BTKi needed further treatment (Fig. 2C). One patient received CAR T-cells and underwent six allogeneic stem cell transplants (Supplementary Results).

### The distinct gene expression profile of CLL harboring t(14;19) points to the activation of proliferative pathways and suppression of cell death

To investigate the molecular characteristics of tCLL, we compared the gene expression profiles of 25 tCLL patients with those of 22 oCLL cases and B cells from 9 healthy donors by sequencing their transcriptomes at high depth with Illumina technology (RNA-seq). The clinical and biological features of the 25 sequenced tCLL patients and their outcomes were similar to those of the entire cohort of 101 patients (Supplementary Table S3; Supplementary Fig. S3A).

**Fig. 3:**
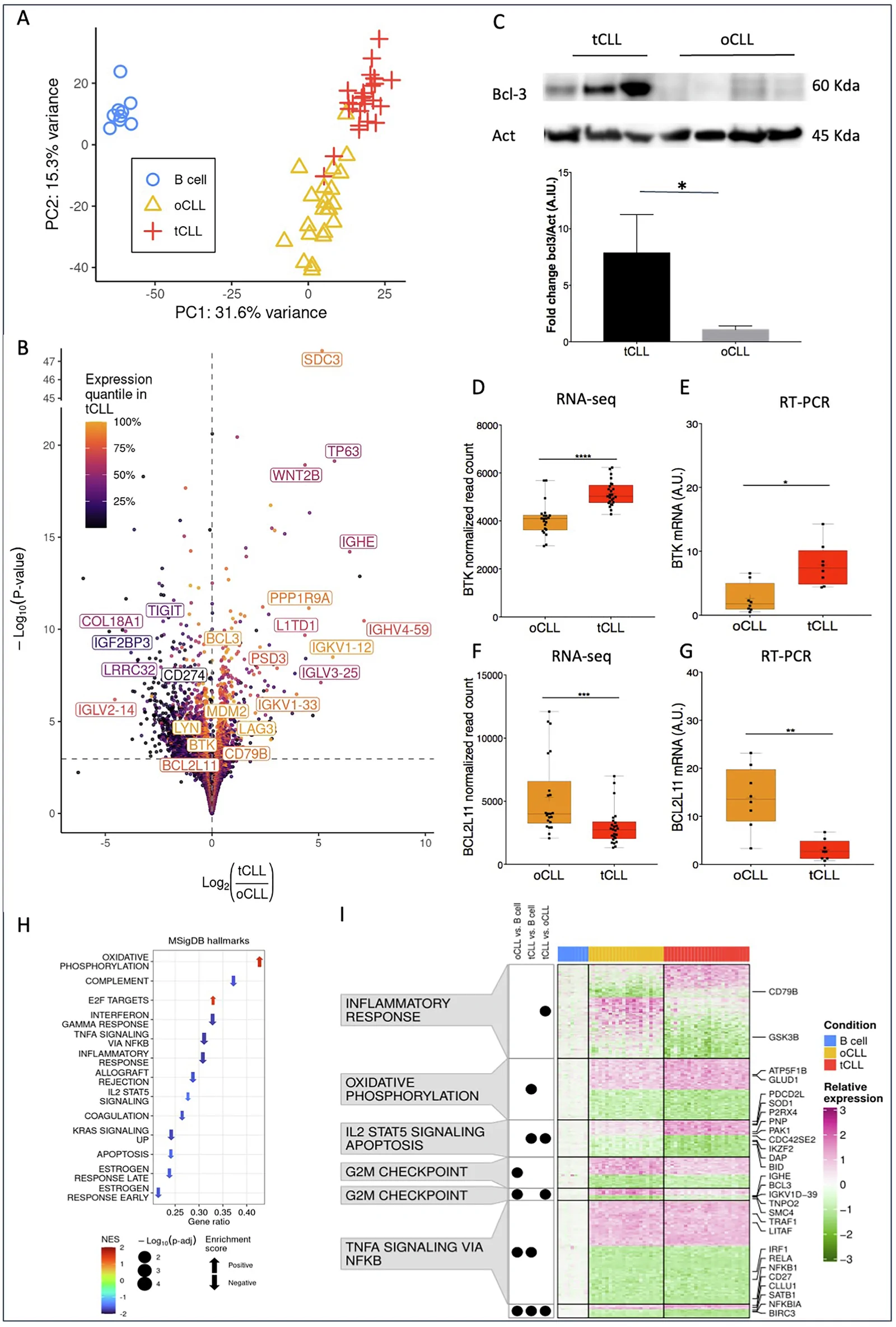
Gene expression profiling of tCLL, oCLL, and B cells from RNA-seq. **A** Dimensionality reduction by principal component analysis (PCA) performed on gene expression estimates. t(14;19) CLL patients (red crosses), control CLL (yellow triangles), and healthy donor B cell (blue circles) samples are displayed according to the first two principal components (PC1, horizontal axis; PC2, vertical axis) explaining the expression variability of the data (31.6% and 15.3%, respectively). **B** Volcano plot of the gene expression differences computed between t(14;19) CLL (tCLL) and other CLL without t(14;19) (oCLL). Dots and labels are colored according to the gene expression rank in tCLL (100% for the most expressed, 0% for the least expressed). **C** Western blotting analysis of Bcl-3 protein in tCLL and oCLL. The histogram in the bottom panel shows the densitometric analysis of Bcl-3/Actin, normalized to normal B cells, expressed as arbitrary units (A.I.). Expression levels, quantified by RNA-seq and RT-qPCR (DDCt method; GAPDH used as reference gene; Mean ± SD shown; A.U., Arbitrary Units) of BTK (**D**, **E**) and BCL2L11 (**F**, **G**) in patients with tCLL and oCLL. **H** Significant gene sets resulted from gene set enrichment analysis on the MSigDb hallmarks. Upward and downward arrows represent positive and negative normalized enrichment scores (NES), respectively, colored according to the magnitude of the NES (red indicates high positive, and blue indicates low negative). The arrow size indicates the statistical significance of the enrichment. **I** Heatmap of differentially expressed genes (rows) between t(14;19) CLL (tCLL), other CLL without t(14;19) (oCLL) samples, and B cells (columns). Pink and green cells represent gene-scaled expression, higher and lower than the gene mean of B cells, respectively. Columns are clustered according to the relative expression; rows are grouped according to differential expression significance, considering the three comparisons oCLL vs. B cells, tCLL vs. B cells, and tCLL vs. oCLL (dots in the panel mid sections indicate the significant comparisons). For each gene set, the enriched pathways are shown on the left side of the panel.

Unsupervised gene expression analysis (Fig. 3A) highlighted distinct profiles between CLL and normal B cells. Additionally, it revealed distinct signatures between tCLL and oCLL samples, with consistent expression patterns among patients within the same group.

By comparing tCLL with oCLL (Supplementary Results), we confirmed the upregulation of the *BCL3* mRNA (LFC = 0.9, p-adjusted <0.001; Fig. 3B; Table 2; Supplementary Fig. S3B), as expected from the t(14;19)(q32;q13) translocation [15], and observed an enrichment of its targets among the overexpressed genes (*p* < 0.001; Supplementary Results). This finding was supported by the observed overexpression of the BCL3 protein, as confirmed through Western blot analysis (Fig. 3C; Supplementary Results).

**Table 2:** Differentially expressed genes between tCLL and oCLL with known relevant roles in CLL.

Gene symbol	Expression rank			Log_2_ fold change			p-adjusted		
	tCLL	oCLL	B cells	tCLL vs. oCLL	tCLL vs. B cells	oCLL vs. B cells	tCLL vs. oCLL	tCLL vs. B cells	oCLL vs. B cells
*BCL2L11*	0.78	0.88	0.93	–0.63	–1.28	–0.33	0.006	0.003	0.257
*BCL3*	0.92	0.82	0.91	0.92	–0.02	–0.87	<0.001	0.961	0.002
*BTK*	0.91	0.88	0.94	0.31	–0.50	–0.83	0.001	0.002	<0.001
*CD79B*	0.86	0.77	0.87	0.67	–0.05	–0.60	0.002	0.925	0.075
*CD274 (PD-L1)*	0.06	0.23	0.12	–1.72	–0.54	0.27	<0.001	0.203	0.278
*LAG3*	0.96	0.87	0.71	1.37	6.32	4.65	0.001	<0.001	<0.001
*LYN*	0.97	0.98	0.99	–0.32	–0.90	–0.55	0.001	<0.001	0.001
*TIGIT*	0.37	0.59	0.17	–1.80	2.48	4.40	<0.001	<0.001	<0.001

Comparison with normal mature B cells is also shown. tCLL: CLL harboring t(14;19); oCLL: other CLL cases, without t(14;19); Expression rank: gene ranking as expression level percentile in tCLL, oCLL, and B cells: 0 =  lowest expression, 1 = highest expression.

Noteworthy was the significantly higher expression of the *CD79B* and *BTK* genes in tCLL (Fig. 3B; Table 2; Supplementary Fig. S3B, Supplementary Fig. S6A), which are key elements of the BCR pathway. Among the differentially expressed immune checkpoint genes, the expression of *PD-L1* and *TIGIT* was decreased, while *LAG3* was highly expressed in tCLL (Fig. 3B, Supplementary Fig. S4, Supplementary Fig. S6B). Genes with high abundance in oCLL that were dramatically downregulated in tCLL included *COL18A1, IGF2BP3*, and *LRRC32* (Fig. 3B). We also observed downregulation of the *BCL2L11* gene (Table 2; Fig. 3B, D–G; Supplementary Figs. S3, S5A), which codes for the pro-apoptotic protein Bim.

Gene set enrichment and pathway analysis revealed that the oxidative phosphorylation (OXPHOS), E2F targets (Fig. 3H), and immunoglobulin complex (Supplementary Figs. S4, S6) were enriched in genes overexpressed in tCLL. Moreover, we observed the downregulation of genes in key pathways related to the suppression of immune processes, chemotaxis, and cytokine signaling (Supplementary Results, Supplementary Table S5, and Supplementary Fig. S7).

Topology-based pathway enrichment analysis [39] (TBA), which considers the interactions and functional outcomes of genes within pathways, indicated the activation of key cancer hallmark signaling pathways, including the PI3K-Akt, B-cell receptor, MAPK, and T-cell receptor signaling pathways (Supplementary Results, Supplementary Table S7, Supplementary Fig. S9).

### Gene expression signature and pathway alteration patterns unique to tCLL

Comparison with normal B cells further clarified the distinctive dysregulation of gene expression and pathway activity in tCLL (Fig. 3I; Supplementary Results).

Interestingly, many genes deregulated in oCLL were not affected in tCLL compared to normal B cells (Supplementary Fig. S3B); these genes were primarily involved in cell cycle regulation, including *BCL3*, *CD79B*, *TNPO2*, *SMC4*, *CENPE*, *AURKB*, and *UBASH3B* (Fig. 3I; Supplementary Table S8). Genes differentially expressed uniquely in tCLL, which can be considered tCLL-related aberrancies, included lower expression of apoptosis regulators and important members of the IL-2/STAT5 signaling pathway, as well as overexpression of pro-proliferative genes (Fig. 3I; Supplementary Results). Accordingly, the ‘inflammatory response’ and multiple signaling pathways (TNFα, IL-2/STAT5, and IL-6/JAK/STAT3) were more downregulated in tCLL than oCLL, suggesting reduced immune capacity and altered cytokine responses. The interferon-gamma response was uniquely downregulated in tCLL, as well as the ‘UV response DN’ (Supplementary Figure S10), which suggests differences in the DNA damage response. Notably, mitotic spindle and G2M checkpoint pathways were downregulated in oCLL but not in tCLL, indicating more active proliferation in tCLL. Finally, the ‘chemokine signaling’ and ‘proteoglycans in cancer’ pathways were significantly activated only in tCLL (Supplementary Fig. S11).

### Patients with tCLL showed a longer TTNT with BTKi than with venetoclax-based therapy

The distinct expression profile and cell state witnessed by the particular landscape of pathway activation in tCLL patients may influence the duration of response to treatment. Notably, reduced BCL2L11 expression can affect the duration of response after Ven-based therapy^(62)^. We hypothesized that higher levels of BTK might impact the response to BTKi. Therefore, we analyzed TTNT after different treatments.

**Fig. 4:**
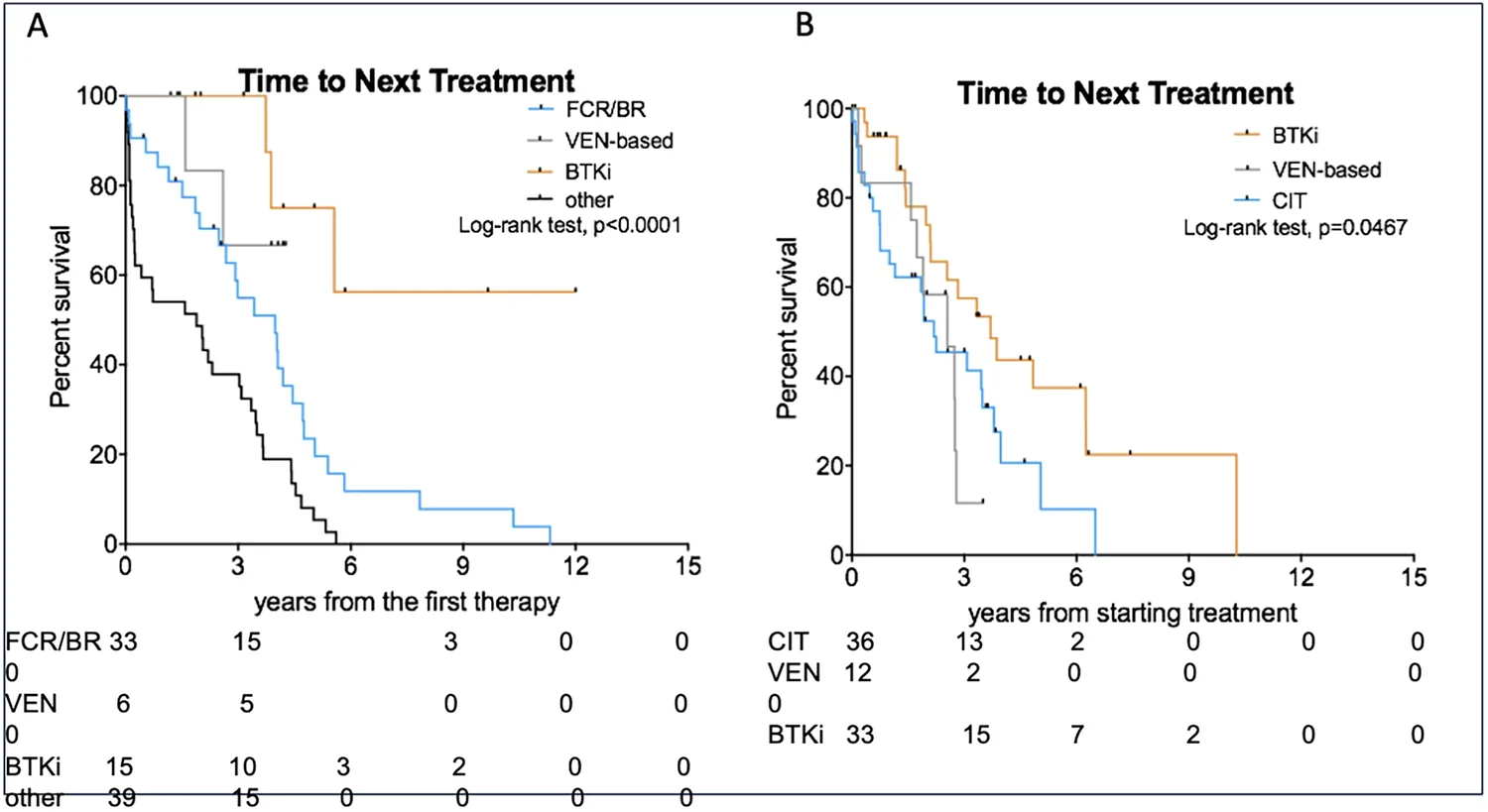
Kaplan-Meier curves for different therapies in patients with tCLL. The Kaplan-Meier curve of time to next therapy after different treatments, such as FCR/BR, venetoclax-based therapy (Ven-based), BTKi-based, or other chemo/chemo-immunotherapy (CIT), both as first-line (**A**) or further-line (**B**) therapy.

The median TTNT was 3.98 and 1.89 years for tCLL patients treated with FCR/BR and other chemotherapies, but not reached for those patients treated with BTKi or Ven-based frontline therapy (*p* < 0.0001; Fig. 4A). After 3 years from the start of treatment, 45%, 33%, 0%, and 62% of patients treated with FCR/BR, Ven-based therapy, BTKi, and other therapies, respectively, needed a second line of treatment (Fig. 4A). In particular, the TTNT was longer in patients treated with BTKi than FCR/BR (*p* = 0.0062, HR 5.19, 95% CI [2.4–7.9]), and there was a trend between BTKi vs. Ven-based therapy (*p* = 0.058, HR 1.7, 95% CI [1.01–12.99]).

Regarding relapsed/refractory patients, 36 (44%) received chemo/chemoimmunotherapy (i.e., chlorambucil ± rituximab, BR, rituximab, etc.), 33 (41%) a BTKi (27 ibrutinib, 5 acalabrutinib, 1 zanubrutinib), and 12 (15%) a Ven-based therapy. The median TTNT was 2.19, 2.55, and 3.7 years for patients treated with CIT, Ven-based therapy, and BTKi, respectively (*p* = 0.0467; Fig. 4B). After 3 years, the rates of patients who started further treatment were 55%, 88% and 42%, respectively (Fig. 4B). The TTNT was significantly longer in patients treated with BTKi than those treated with CIT (*p* = 0.0253, HR 1.89, 95% CI [1.03–3.46]). However, it also tended to be more prolonged in patients managed with a Ven-based therapy, although this did not reach statistical significance (*p* = 0.1051, HR 2.15, 95% CI [0.96–5.64]).

In summary, tCLL patients showed durable responses if treated with continuous BTKi but experienced early relapse if treated with chemoimmunotherapy or with Ven-based time-limited therapy.

## Discussion

In this study, we characterized the clinical and transcriptomic features in a large cohort of patients with CLL harboring the rare t(14;19) (tCLL), thanks to an extended international collaboration. We report evidence suggesting that identifying t(14;19), although a rare entity, by CBA in the era of continuous and fixed-duration therapy for CLL may help in tailoring treatment. Although karyotyping is now recommended by some CLL guidelines [1, 2] to identify patients with more than 3 or 5 chromosomal abnormalities (i.e., those harboring a CK) for treatment selection, its role remains controversial. However, it should not be limited to the number of chromosomal abnormalities, as some specific chromosomal rearrangements or lesions may have a particular prognostic or predictive impact. It is well known that patients harboring trisomies (i.e., +12, +19, +other numerical or structural aberrations) have a favorable outcome despite having a complex karyotype [7]. In our cohort, we demonstrated that patients with tCLL have an overall unfavorable prognosis independent of a complex karyotype, whether it is CK3 or CK5.

Most published studies on patients with tCLL reported an atypical immunophenotype and morphology with an increased frequency of trisomy 12, U-IGHV genes, and overexpression of the *BCL3* gene [13–21]. We confirmed all these features, including the overexpression of the BCL3 protein. We also described a high rate of BCR stereotype #8 in tCLL, associating a chromosomal translocation with a specific stereotyped BCR. The transcriptomic analysis found that the *TP63* gene, a member of the p53 transcription factor family, was overexpressed in our cohort of patients with tCLL. In line with our data, a previous study indicated that the *TP63* gene was hypomethylated and overexpressed in stereotyped subset #8 [14, 41].

RNA sequencing enabled us to identify a distinct gene expression profile of tCLL compared to oCLL. In particular, we found a higher expression of the *CD79B* gene and protein, which justifies the atypical immunophenotype of the cells. Additionally, IGHG4 was overexpressed, leading to the expression of IgG on the surface of CLL cells. IgG–switched CLL is a rare variant of CLL that displays an overall distinct immunogenetic signature [42]. Notably, IgG-switched CLL is enriched in stereotype BCR #8 with an unmutated status of the IGHV gene and trisomy of chromosome 12 [42]. In our patient cohort, almost one-third of tCLLs were IgG-switched.

Among the most differently activated pathways, we found that the immune checkpoints TIGIT and PD-L1 were downregulated, while LAG3 was, conversely, overexpressed in tCLL. The dysregulated expression of immune checkpoints [41–44] and the low efficacy of the PD1 inhibitors have already been reported in CLL [45, 46]. Shapiro et al [47]. demonstrated a higher surface LAG3 expression associated with U-IGHV and a shorter time to first treatment. Interestingly, LAG3-blocking antibodies enhance in vitro T-cell activation [47]. The TIGIT pathway has also been studied in CLL [48], and its expression was inversely correlated with time to first treatment and the B cell receptor (BCR) signaling capacity, as determined by BTK activity and cell proliferation [48]. Along this line, a low baseline level of PD-L1 and TIGIT by tumor cells has been reported in patients with large B-cell lymphoma who have shown a better response to CAR T cells [44]. While these findings suggest potential clinical relevance, the exact roles of TIGIT and LAG3 in tCLL pathogenesis and treatment response require further investigation. The use of LAG3-blocking antibodies [49] and CAR T cells represents intriguing directions for future research but needs thorough clinical evaluation to determine their efficacy in tCLL.

Oxidative phosphorylation (OXPHOS) and E2F target pathways [50] were activated, whereas apoptosis-related pathways were suppressed specifically in tCLL. Furthermore, cancer hallmark signaling pathways, including the PI3K-Akt, B-cell receptor, MAPK, and T-cell receptor signaling pathways, were activated. While it is already known that CLL cells primarily use OXPHOS for generating energy [51], the OXPHOS enrichment in tCLL suggests higher oxidative stress related to an abundant generation of reactive oxygen species (ROS) by increased mitochondrial production [52], which can be linked to a more proliferative and aggressive phenotype in tCLL.

The gene expression comparison of the two CLL groups to normal B cells allowed us to highlight distinctive molecular characteristics for tCLL. Intriguingly, our data suggest possible mechanisms that could contribute to regulating the *BCL3* abundance in tCLL, such as the GSK3β-mediated protein degradation pathway [53, 54] and miR-181a targeting BCL3 [55–59]. The GSK3β-mediated protein degradation is supported by the *GSK3B* expression level, which was very low in tCLL. Regarding miR-181a-mediated regulation, the high BCL3 abundance in tCLL matched with a lower miR-181a expression compared to B cells. Interestingly, tCLL displayed a specific upregulation of PAK1, previously shown to promote proliferation and sustain ibrutinib resistance in CLL.

Our results suggest that the translocation-induced overexpression of the *BCL3* gene in tCLL triggers a complex and widespread dysregulation of gene expression. We identified molecular alterations specific to tCLL, which involved several cancer hallmarks signaling pathways (TNF-α, IL-2/STAT5, IL-6/JAK/STAT3, and PI3K-AKT) and metabolic processes that may affect apoptosis, cell proliferation, survival, antibody production, response to the microenvironment, homing and migration, and potentially drug resistance. Overall, this might help explain the worse outcome of patients with tCLL compared to those with oCLL. Notably, we demonstrated that circular RNAs are commonly dysregulated in tCLL, exhibiting a specific signature whose biological significance warrants further investigation [60].

A limitation of this study is its retrospective observational design, which encompasses over 20 years. During this timeframe, chromosome banding analysis was only recommended, not mandatory, and was therefore performed in only a few selected centers. For this reason, the prevalence of patients with tCLL might be inaccurate. To minimize selection bias and inaccurate data reporting, we asked physicians to report all consecutive patients with CLL harboring t(14;19) tested within 1 year of diagnosis. In addition, despite only a few patients receiving frontline Ven-based therapy, a statistical difference in the TTNT was found. Additionally, there may be biases influencing the samples collected for the transcriptomic analysis. However, as reported here, the TTFT and OS rates of the patients selected for RNA-seq did not significantly differ from those of the overall cohort, supporting the representativeness of our selection.

Furthermore, when comparing the TTNT between patients with tCLL or oCLL, we only included individuals who received similar treatments to minimize bias from differing therapy regimens. It should also be acknowledged that t(14;19) has been associated with a broad spectrum of B lymphoid neoplasms. Carbo-Meix A. et al. [14] recently reported that *BCL3* breakpoints occur in two clusters at 5ʹ (*n* = 9) and 3ʹ (*n* = 4) regions of *BCL3*. While both breakpoints were mediated by aberrant class switch recombination of the IGH locus, only the 5’ breakpoints juxtaposed *BCL3* upstream to an IGH enhancer, leading to the overexpression of the *BCL3* gene. They found that the majority of upstream *BCL3*-rearranged tumors had unmutated IGHV, trisomy 12, an atypical CLL morphology, immunophenotype, DNA methylome, and expression profile that differ from CLL without t(14;19). For these reasons, we included only patients with a Matutes score of 3 or higher, mainly due to the expression of CD5, CD23, and higher expression of CD79b and sIg. Our study, which included only CLL with t(14;19), yielded similar results to those found in upstream *BCL3*-rearranged tumors.

By both RNA-seq and RT-PCR, we observed that tCLL was characterized by higher levels of *BTK* and the downregulation of the *BCL2L11* gene, which encodes the pro-apoptotic protein BIM. Accordingly, the time to second-line therapy with a time-limited venetoclax-based therapy was shorter in patients with tCLL compared to those with oCLL, likely due to low mitochondrial priming and lower sensitivity to BCL2 inhibition, thereby impeding sustained remission. Conversely, continuous BTKi provides sustained remissions. It is noteworthy that in the exploratory post-hoc analyses of the CLL14 trial, which investigated venetoclax-obinutuzumab in elderly patients with CLL, achieving undetectable measurable residual disease (i.e., uMRD6) status was associated with higher pro-apoptotic *BCL2L11* expression [61]. Furthermore, as in our study, the PI3K-Akt signaling pathway has been linked to *BCL3* overexpression [62]. While targeting PI3K might be an alternative option for this subset of patients with CLL, the clinical use of PI3K inhibitors is limited by a wide range of adverse events [63].

## Conclusions

In conclusion, we herein reported that patients with tCLL are younger than those with oCLL, enriched for +12, CK, U-IGHV, and stereotyped subset #8, and have a distinct gene expression profile featured by higher expression of *BTK* and lower apoptotic priming due to low *BCL2L11* levels, driven by the overexpression of the *BCL3* gene. In our retrospective cohort, continuous BTKi seems to be more effective than a time-limited strategy. Although a few compounds have recently been identified and tested in vitro [64–66], no clinically staged BCL3 inhibitor is currently available. Further biological and molecular studies are warranted to better understand the aggressive CLL that harbors the t(14;19) translocation .

## Supplementary information

Supplementary materials and results

Supplementary Figures

Supplementary Tables

## Data Availability

PRO-Seq data were deposited into the ArrayExpress repository under accession number E-MTAB-15004.
