# Supplementary material for: Clinical and transcriptomic characterization of patients with chronic lymphocytic leukemia harboring t(14;19): an ERIC study
Source: Leukemia. 2025 Sep 19;39(12):2957–67. doi: 10.1038/s41375-025-02755-8 (PMC12634426; doi:10.1038/s41375-025-02755-8)
Supplement: Supplementary file 1 — Supplementary materials and results [file 41375_2025_2755_MOESM1_ESM.docx]

Clinical and transcriptomic characterization of patients with chronic lymphocytic leukemia harboring t(14;19): an ERIC study

*Supplementary Information*

#

[**Supplementary Methods 2**](#_heading=h.ymdappehovt4)

Cells isolation 2

[IGHV mutational status 2](#_heading=h.6whwg3b77h0t)

[Cytogenetics by fluorescence in situ hybridization (FISH) and TP53 mutation 2](#_heading=h.379crrgsswjv)

[Quantification of gene expression by RT-qPCR](#_heading=h.lnxbz9) 3

[Flow cytometry 3](#_heading=h.35nkun2)

[Western Blotting 3](#_heading=h.yaudusf85c5s)

[Statistical analysis 4](#_heading=h.17dp8vu)

[**Supplementary Results 5**](#_heading=h.83uau6uynz0k)

[The distinct gene expression profile of CLL harboring t(14;19) points to the activation of proliferative pathways and suppression of cell death 5](#_heading=h.km2ng0jd5r42)

[Gene expression signature and pathway alteration patterns unique to tCLL 6](#_heading=h.u2aglqi5sjzh)

[**Supplementary Figures 8**](#_heading=h.tx9jaj5djwd)

#

#

# Supplementary Methods

## Cells isolation

Peripheral blood mononuclear cells (PBMCs) of the patients with CLL were isolated by density-gradient centrifugation over Lymphoprep (STEMCELL Technologies; Vancouver, Canada). When the fraction of neoplastic cells (CD19+/CD5+) was less than 97%, an additional purification was performed with the RosetteSep™ B cell enrichment Cocktail (STEMCELL Technologies). Healthy human B cells were purified by negative selection using the RosetteSep™ B cell enrichment Cocktail. Ultimately, all samples used in the study (both from patients and healthy subjects) contained at least 97% of CD19+ cells, as assessed by flow cytometry, thus minimizing contamination from other cell types

## IGHV mutational status

Analysis of the IGHV mutational status was performed within 12 months of diagnosis on peripheral blood CLL cells from fresh samples or frozen, purified CLL cells harvested in DMSO, according to the ERIC guidelines and certification. Sequences were analyzed using the IMGT/VQUEST and BLAST software to detect VDJ junctions. Cases with a sequence homology < 98% from the corresponding germline gene were considered mutated (M-IGHV), and those with a homology 98% as unmutated (U-IGHV). The stereotyped B-cell receptor (BCR) was assessed with ARResT.

## Cytogenetics by fluorescence in situ hybridization (FISH) and TP53 mutation

FISH was performed on standard cytogenetic preparations from peripheral blood according to the GenQA guidelines. The slides were hybridized with the multicolor probe set LSI p53/LSI ATM and LSI D13S319/LSI 13q34/ CEP12 and RP11-177O8 according to the manufacturer’s instructions. One to three hundred interphase nuclei were analyzed for each probe. TP53 gene sequencing was performed according to the ERIC 2018 guidelines, assessing exons 4-10. If negative, exons 2, 3, and 11 were also investigated.

## Quantification of gene expression by RT-qPCR

Total RNA was extracted with the RNeasy Mini Kit (Qiagen, Hilden, Germany) and treated with DNase (Qiagen), according to the manufacturer’s protocol. Complementary DNA was generated from 1 mg of total RNA using random primers and the AMV reverse transcriptase (Promega). The quantification of selected gene transcripts was assessed by real-time quantitative PCR (RT-qPCR) in a cohort of patients. RT-qPCR was performed with the Luna® Universal qPCR Master Mix (NEB) and a QuantStudio™ 5 Real-Time PCR System (ThermoFisher Scientific). The relative expression of transcripts was normalized to GAPDH expression and determined with the DDCt method. Primer sequences are listed in Supplementary Table S1.

## Flow cytometry

Aliquots of 250x10^5^ purified CLL cells were harvested, washed in PBS 1x and incubated for 10 min in the dark at room temperature and co-stained with the following antibodies: CD274 (PE-Cy7 conjugated, CD79b (FITC conjugated), CD223 (LAG-3) (PE-conjugated), TIGIT (PE-conjugated, Becton Dickinson; Franklin Lakes, NJ, USA). After incubation with antibodies, cells were washed with PBS and analyzed by flow cytometry. For each sample, 20,000 events were acquired and analyzed using the FACSCanto II^TM^ A cytometer, and data were processed using DIVA Software (Becton Dickinson). The mean fluorescence intensity was reported for each antibody in comparison to the untreated condition.

## Western Blotting

Whole-cell extracts were obtained by RIPA Lysis and extraction buffer (Tris-HCL 20mmol/l, NaCl 150mmol/l, EDTA 5.0mmol/l, Niaproof 1.5%, Na3VO4 1.0mmol/l, SDS 0.1%, Thermo Fisher Scientific, Waltham, MA, USA), added with protease inhibitors (Halt Protease Inhibitor Cocktail, Thermo Fisher Scientific), phosphatase inhibitors (Phosphatase Inhibitor Cocktail, Thermo Fisher Scientific), and EDTA (Thermo Fisher Scientific) on ice. The supernatant was quantified by a BCA protein quantification assay (Thermo Fisher Scientific). Equal amounts of protein sample were added to 3 × Red loading sample buffer (Cell Signaling Technology, Danvers, USA) and boiled for 5 min. Western blotting was conducted according to standard protocols. Briefly, 15 to 25 μg of whole-cell extracts were subjected to SDS-PAGE, transferred to Nitrocellulose/PVDF membranes, and immunoblotted with the following primary antibodies: Bcl3 antibody (Cell Signaling Technology, Danvers, MA) and anti-β-actin (Sigma-Aldrich, St. Louis, MO). Detection was performed using a chemiluminescence reaction (ECL, Euroclone, Milan, Italy). Images were acquired using the Amersham Imager 600 (GE Healthcare; Chicago, IL, USA), and the protein bands were scanned and quantified by densitometry using the ImageJ program (Github, San Francisco, USA).

## Statistical analysis

Categorical variables were compared using the Chi-square test (for Binet stages and FISH) or Fisher’s exact test (gender, treatment, *TP53,* and *IGHV status*) when appropriate. The median age was compared with the Mann-Whitney test. TTFT was calculated starting from the date of diagnosis to treatment (event) or last known follow-up (censored); TTNT was calculated as years from first-line therapy to a subsequent treatment or last known follow-up; OS was calculated starting from the date of diagnosis to death for any cause or last known follow-up. Survival analyses were performed using the Kaplan-Meier method, and the Log-rank test was used to compare survival curves between groups. The Cox regression model was employed to estimate hazard ratios (HR). A p-value less than 0.05 was considered significant. Statistical analysis was performed with the R programming language.

#

# Supplementary Results

## Details on patients who received allogeneic stem cell transplantation and CAR T.

Only one patient received CAR T cells within a clinical trial after undergoing four lines of therapy. However, the patient died after 21 months.

Among 101 patients, 6 received allogeneic stem cell transplantation, and their outcomes as follows:

1. He received it as consolidation after FCR, and the remission lasted 8 years, then he received 3 lines of therapy and subsequently he died;
2. He did it after 4 lines of therapy, relapsed after 2 years, received other 5 therapies and then died;
3. She did allogeneic stem cell transplantation after 4 lines (FCR, venetoclax-rituximab, ibrutinib, idelalisib-rituximab), and she is alive and still in remission after 4 years;
4. She did it after 2 lines of therapy (FCR and Ibrutinib) and is still in remission after 5 years;
5. She did it after 2 lines of chemotherapy, but died after 3 months due to infection;
6. He received it after 3 lines of therapy (including FCR, ibrutinib and venetoclax) after 11 months, and he is still in remission.

##

## The distinct gene expression profile of CLL harboring t(14;19) points to the activation of proliferative pathways and suppression of cell death

Differential expression analysis identified 737 genes with higher expression in tCLL compared to oCLL and 1,270 genes with lower expression in tCLL (Figure 3B, Supplementary Table S4).

According to the TFLink database^1^, 66% of the genes overexpressed in tCLL could be targets of the BCL3 transcription factor(p<0.0001; Supplementary Figure S3). We identified potential targets of the BCL3 transcription factor within the most significantly overexpressed and abundant genes in tCLL, including *MDM2*, *CD79B,* and the oncogenes *WNT2B* and *PTPN6* (Figure 3B; Supplementary Table S4). Interestingly, the *TP63* transcription factor, which targets *BCL3*, was dramatically upregulated (LFC = 5.7) and abundant in tCLL, whereas it was barely expressed in oCLL (Supplementary Table S4).

The differential expression levels of selected genes, including *BTK*, *BCL2L11*, *LYN*, *LAG3*, *MDM2*, and *PTPN6*, were confirmed by RT-PCR (Supplementary Figure S5A). In addition, the protein expression levels of CD79B, LAG3, PD-L1 and TIGIT, measured by flow cytometry compared to oCLL and healthy B cells, confirmed our observations based on RNA-seq (Supplementary Materials; Supplementary Figure S5B).

To better characterize the cell state differences in tCLL compared to oCLL, we investigated whether specific sets of genes or pathways were differently modulated in the two CLL groups by performing gene set enrichment analysis^2^ (GSEA) on multiple gene signatures, including MSigDB[^3,4^](https://www.zotero.org/google-docs/?broken=73SDIT), Gene Ontologies, KEGG, Reactome, and WikiPathways (Supplementary Table S4).

Interestingly, the OXPHOS signature was enriched in genes overexpressed in tCLL and significantly enriched in genes that are BCL3 targets (Supplementary Figure S3C; Supplementary Table S6), suggesting a role of BCL3 overexpression in sustaining metabolic changes in tCLL. All significantly enriched Gene Ontology terms and pathways were linked to genes downregulated in tCLL (Supplementary Figure S6; Supplementary Table S4).

Topology-based pathway enrichment analysis[^4^](https://www.zotero.org/google-docs/?broken=pFu2hM) (TBA) revealed that tCLL presented inhibition of the Ras, VEGF, Rap1, calcium, and ‘chemokine signaling pathways and cytokine-cytokine receptor interaction’ (Supplementary Table S7).

## Gene expression signature and pathway alteration patterns unique to tCLL

A comparison of the tCLL and oCLL to B cells further clarified the distinctive gene expression and pathway activity dysregulation in tCLL, as well as underscored common features.

Compared to B cells, 1,245 genes were upregulated, and 1,519 genes were downregulated in tCLL. In oCLL, 963 genes showed higher expression, while 1,092 showed lower expression compared to B cells.

Genes similarly altered in tCLL and oCLL, such as the upregulated *TRAF1* and *LITAF*, and many commonly downregulated genes, including *NFKB1/2*, *REL*, *RELA*, *NFKBIE*, and *IRF1*, highlighted the modulation of TNFα signaling via NF-κB pathway in both CLL types (Figure 3I).

We further investigated the effect of *BCL3* translocation by comparing the abundance of BCL3 protein in B cells by Western blotting. Unexpectedly, BCL3 protein was barely detected in B cells despite the *BCL3* transcript expression being comparable to tCLL (Supplementary Figure S7).

The genes altered uniquely in tCLL, which can be considered tCLL-related aberrancies, included apoptosis regulators such as the downregulated pro-apoptotic *BID*, *DAP*, *TNFSF10*, *PDCD4*, and *BCL2L11* (Supplementary Figure S5), as well as the upregulated oncogenes *SOD1,* counteracting oxidative damage, and *PAK1*. Additionally, important members of the IL-2/STAT5 signaling pathway were altered specifically in tCLL, including *IKZF2*, whose downregulation can contribute to uncontrolled proliferation and apoptosis escape in B cells. Instead, the pro-proliferative *PDCD2L* and *P2RX4* genes were upregulated only in tCLL, as were *CDC42SE2*, *PLSCR1*, *PNP*, and *LTB* genes (Supplementary Figure S6; Supplementary Table S8).

We identified 45 genes that showed significantly higher expression in oCLL compared to normal B cells, with even greater expression levels in tCLL. These included *CLLU1*, *PEBP1*, *LBH*, *RASSF3*, the receptors *CD27* and *LAG3* (Supplementary Figure S5), and the oncogenic kinase *RIOK2*, a potential therapeutic target. Conversely, we found 102 genes exhibiting significantly reduced expression in oCLL compared to B cells, with even more marked reductions observed in tCLL. These included genes involved in B-cell lineage development (e.g., *SATB1*), the NF-κB inhibitor *NFKBIA*, the anti-apoptotic *BIRC3*, the transcriptional regulator *ZBTB10*, and the miR-181 host gene *MIR181A1HG*.

GSEA revealed that when comparing tCLL or oCLL versus B cells, most pathways were significantly enriched in genes downregulated in both CLL types, with more pronounced effects in tCLL (Supplementary Figure S8).

Hypoxia pathway downregulation differed between the CLL subtypes, indicating varied adaptation to low oxygen (Supplementary Figure S9).

Further considering pathway activation, TBA revealed distinct pathways inversely modulated in the two groups compared with B cells (Supplementary Figure S10). The Rap1, T cell receptor, and calcium signaling pathways, inhibited in tCLL, were activated in oCLL. In addition, several pathways were more impacted in tCLL than oCLL, as indicated by an increased TBA significance score, including the pathway ‘microRNAs in cancer’ (Supplementary Figure S10).

**Supplementary references**

1. [Liska, O. *et al.* TFLink: an integrated gateway to access transcription factor–target gene interactions for multiple species. *Database* **2022**, baac083 (2022).](https://www.zotero.org/google-docs/?broken=96Ruad)
2. [Liberzon, A. *et al.* Molecular signatures database (MSigDB) 3.0. *Bioinforma. Oxf. Engl.* **27**, 1739–1740 (2011).](https://www.zotero.org/google-docs/?broken=lTKWWD)
3. [Liberzon, A. *et al.* The Molecular Signatures Database (MSigDB) hallmark gene set collection. *Cell Syst.* **1**, 417–425 (2015).](https://www.zotero.org/google-docs/?broken=kqJKae)
4. [Tarca, A. L. *et al.* A novel signaling pathway impact analysis. *Bioinforma. Oxf. Engl.* **25**, 75–82 (2009).](https://www.zotero.org/google-docs/?broken=Fs38iv)

#

# Supplementary Figures

**Figure S1.** Oncoprint plot showing the clinical and molecular characteristics of the 101 CLL patients carrying the t(14;19)(q32;q13) translocation. White cells indicate data not available. Aberrant TP53 “yes” either if TP53 was mutated or deleted. CK: complex karyotype.

**Figure S2.** Kaplan-Maier curves of treatment-free survival and overall survival according to (A) the presence of TP53 abnormalities (TP53 abn), (B) trisomy of chromosome 12 (+12), (C) complex karyotype (CK), and (D) complex karyotype with at least five chromosomal abnormalities (CK5) in patients with CLL harboring t(14;19). P-values of the comparisons are reported in the tables on the right.

**Figure S3.** Treatment-free survival (TFS) and Overall survival (OS) of all patients and those whose samples were studied by RNA sequencing (A). Boxplots of CLL-relevant genes’ expression measured by RNA-seq and grouped according to CLL patients harboring or not t(14;19) (tCLL and oCLL, respectively), and healthy donor B cell samples (B).

**Figure S4. BCL3 target genes.** Bar plots of the (A) number and (B) fraction of BCL3 target genes (red portion of the bars) identified among the differentially expressed genes (DEG) estimated by comparing patients with CLL harboring t(14;19) (tCLL), CLL patients without t(14;19) (oCLL) and healthy donors’ B cell samples. (C) Gene set enrichment analysis results on the MSigDB hallmark gene sets (vertical axis) calculated for the BCL3 targets differentially expressed in comparing tCLL, oCLL, and B cells (horizontal axis). Upward yellow-to-red arrows represent positive normalized enrichment scores (NES), and downward green-to-blue arrows represent negative NES scores of the enriched terms. Arrow size is proportional to the ratio of genes over the gene set size (GeneRatio).

**Figure S5. Fold change of immunoglobulin genes comparing tCLL and oCLL.** Boxplots showing the log_2_ expression fold change (FC) of immunoglobulin (IG) genes (void dots) comparing tCLL vs. oCLL separated by chromosome coordinates and colored by IG group. The dot size is proportional to the expression abundance in tCLL, reported as transcripts per million mapped reads (TPM) on a log_10_ scale.

**Figure S6. Validation of the selected genes by RT-PCR and flow cytometry.** Boxplot of the relative gene expression quantified by (A) RNA sequencing, RT-qPCR (DDCt method; GAPDH used as reference gene; Mean ± SD shown; A.U., Arbitrary Units) and (B) flow cytometry in a validation group of patients and normal B-cells samples (at least six samples/group were analyzed for each gene). *, p<0.05; **, p<0.01; ***, p<0.001; ****, p<0.0001.

**Figure S7. Gene set enrichment analysis for the tCLL vs. oCLL comparison.** The significantly enriched gene ontology (GO) biological process (BP), molecular function (MF), and cellular component (CC) terms (vertical axis); in the middle of the central column, the Wiki Pathways (WP), and in the bottom central column, the KEGG pathways. The Reactome terms are on the bottom right panel. Upward and downward arrows represent positive and negative normalized enrichment scores (NES), respectively, which are colored according to the normalized enrichment score (NES), yellow-to-red for positive scores, and green-to-blue for negative scores. The arrow size is proportional to the enrichment significance (-Log_10_(P-adj)). The fraction of the core enrichment gene number over the gene set size (GeneRatio) is plotted on the horizontal axis.

**Figure S8.** Western blotting plot Bcl3 antibody (60Kda) and beta-actin in normal B cells, CLL with (14;19) and without t(14;19) (results of FISH analysis of the control cases are reported on top; NK: CLL with normal karyotype). The histogram below shows the average and standard deviation densitometric analysis of bcl3/actin normalized to normal B cells as arbitrary units (A.IU.).

**Figure S9.** A network of the differentially expressed genes included in the pathways activated in tCLL compared to oCLL. Dots represent genes (pink or cyan, according to the log fold change of the expression comparison) and the corresponding pathways (yellow). Dot size is proportional to the gene abundance in tCLL. Edges connect genes to the pathways in which they are included.

**Figure S10.** Significant gene sets resulted from the gene set enrichment analysis on the MSigDB hallmarks. Downward arrows represent negative normalized enrichment scores (NES) and are colored according to the significance (-log10(P-adj). The arrow size indicates the fraction of the core enrichment gene number over the gene set size (GeneRatio). The dendrogram on the right side illustrates the clustering of terms and pathways based on the similarity of the core enrichment gene sets.

**Figure S11.** Topology-based pathway enrichment analysis of significant cancer pathways. The color scale is proportional to the statistical significance (−log10(p-adjusted)) of the pathways' activation (purple) or inhibition (green) enrichment score. Pathways are ordered by their significance in the comparison of tCLL vs. B cells.
