# Supplementary material for: Clinical and transcriptomic characterization of patients with chronic lymphocytic leukemia harboring t(14;19): an ERIC study
Source: Leukemia. 2025 Sep 19;39(12):2957–67. doi: 10.1038/s41375-025-02755-8 (PMC12634426; doi:10.1038/s41375-025-02755-8)

# Supplementary Figures

Supplementary Figure S1

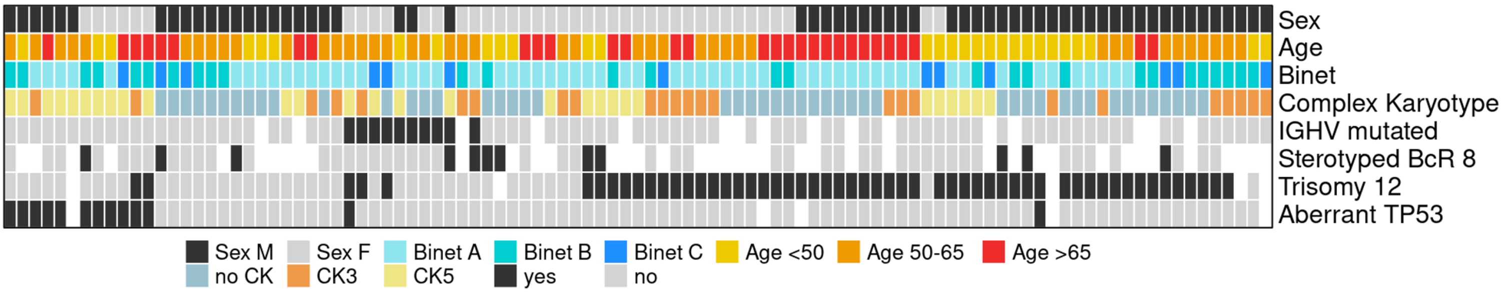

Figure S2

A

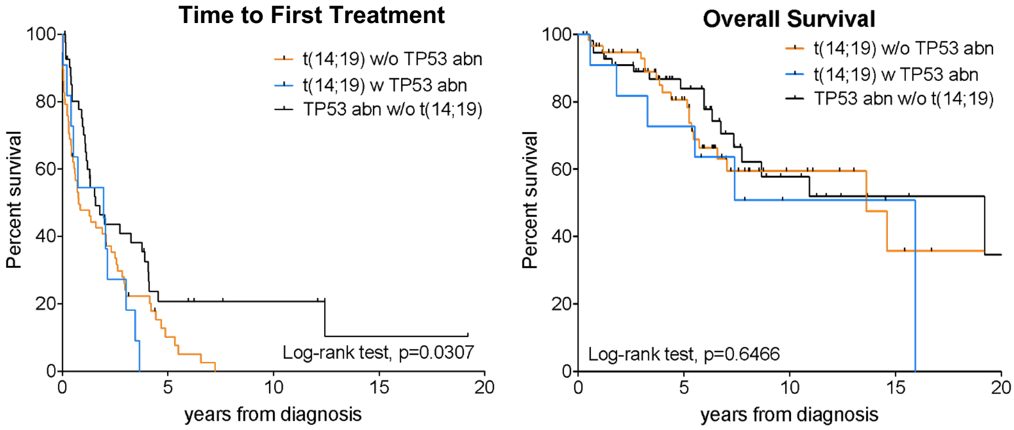


| | t(14;19) w TP53 abn | t(14;19) w/o TP53 abn |
|---|---|---|
| TTFT | | |
| TP53 abn w/o t(14;19) | 0.0637 | 0.0179 |
| OS | | |
| TP53 abn w/o t(14;19) | 0.3074 | 0.6380 |

B

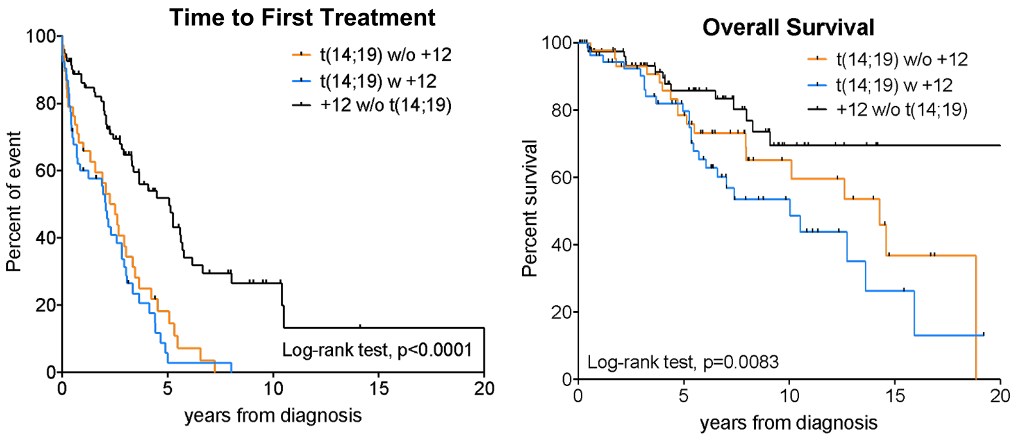


| | t(14;19) w +12 | t(14;19) w/o +12 |
|---|---|---|
| TTFT | | |
| +12 w/o t(14;19) | <0.0001 | <0.0001 |
| OS | | |
| +12 w/o t(14;19) | 0.0318 | 0.2908 |

C

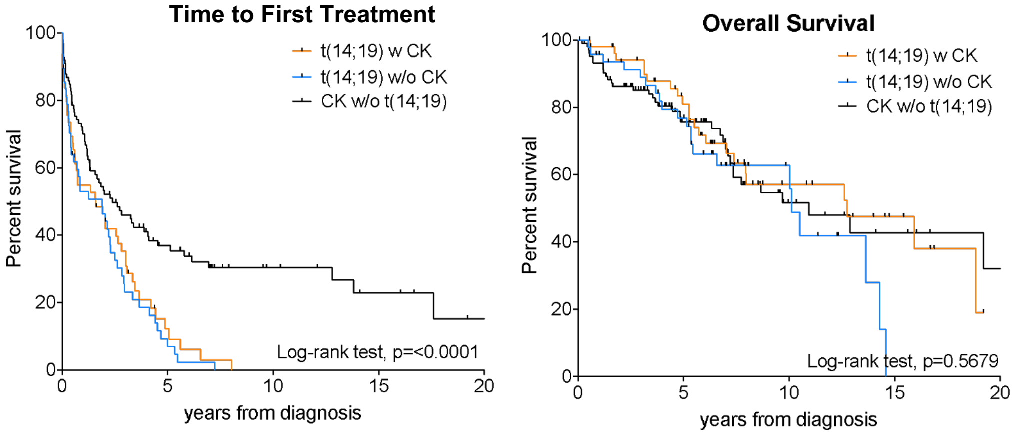


| | t(14;19) w CK | t(14;19) w/o CK |
|---|---|---|
| TTFT | | |
| CK w/o t(14;19) | 0.0012 | 0.0001 |
| OS | | |
| CK w/o t(14;19) | 0.7656 | 0.4726 |

D

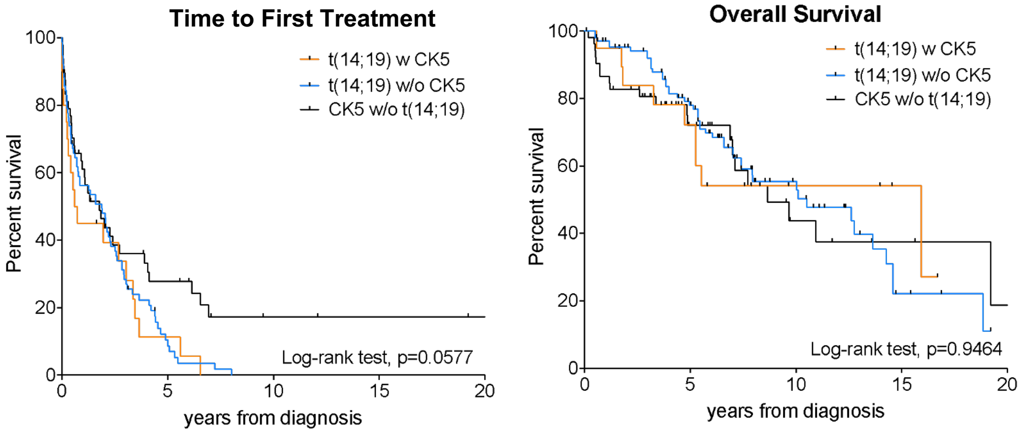


| | t(14;19) w CK5 | t(14;19) w/o CK5 |
|---|---|---|
| TTFT | | |
| CK5 w/o t(14;19) | 0.0658 | 0.0447 |
| OS | | |
| CK5 w/o t(14;19) | 0.9258 | 0.7312 |

Figure S3

A

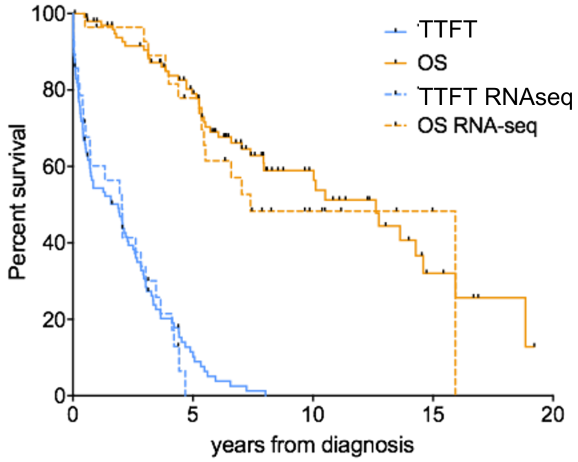
TTFT
OS
TTFT RNAseq
OS RNA-seq
Percent survival
years from diagnosis

B

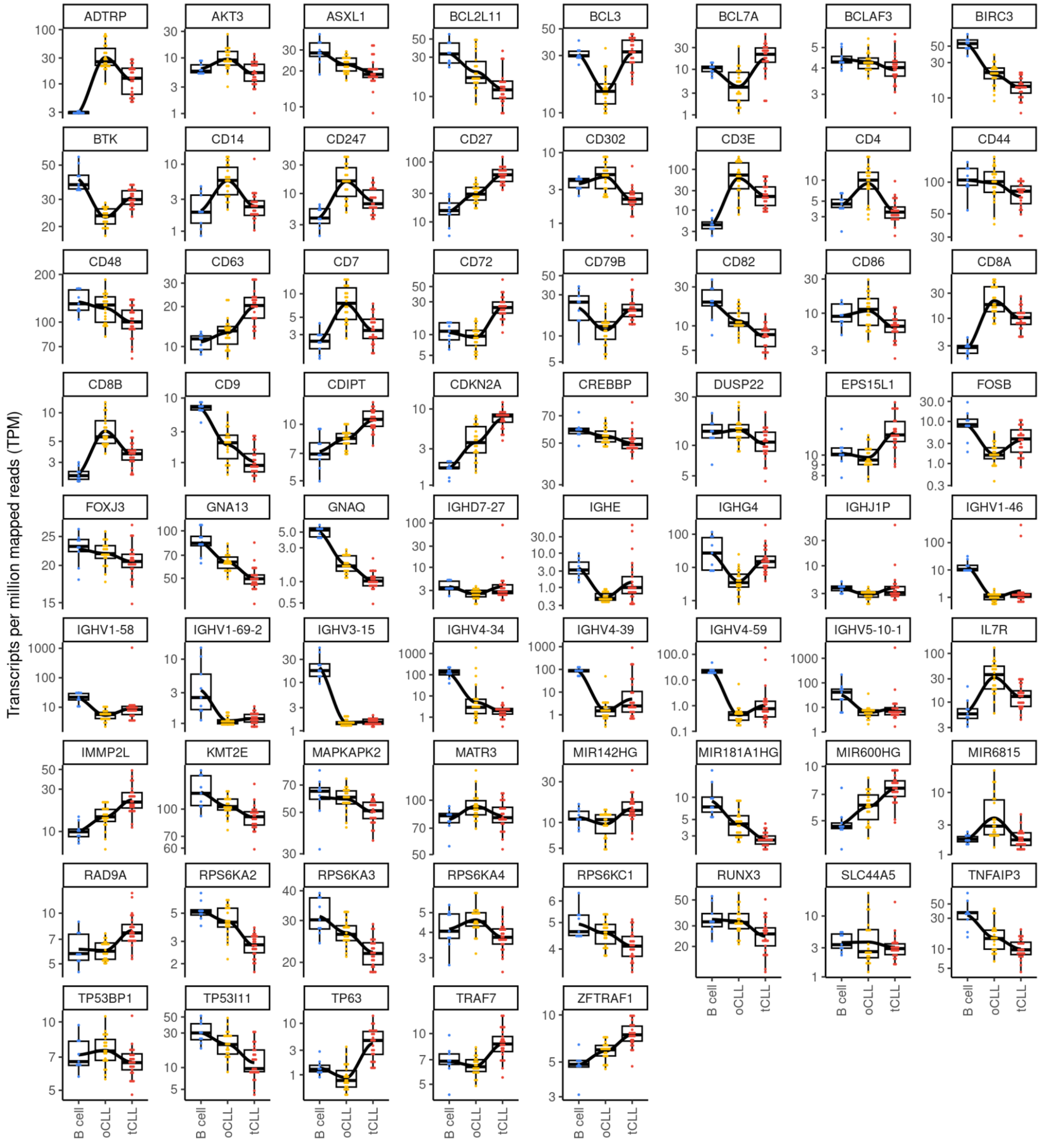
Transcripts per million mapped reads (TPM)
ADTRP
AKT3
ASXL1
BCL2L11
BCL3
BCL7A
BCLAF3
BIRC3
BTK
CD14
CD247
CD27
CD302
CD3E
CD4
CD44
CD48
CD63
CD7
CD72
CD79B
CD82
CD86
CD8A
CD8B
CD9
CDIPT
CDKN2A
CREBBP
DUSP22
EPS15L1
FOSB
FOXJ3
GNA13
GNAQ
IGHD7-27
IGHE
IGHG4
IGHJ1P
IGHV1-46
IGHV1-58
IGHV1-69-2
IGHV3-15
IGHV4-34
IGHV4-39
IGHV4-59
IGHV5-10-1
IL7R
IMMP2L
KMT2E
MAPKAPK2
MATR3
MIR142HG
MIR181A1HG
MIR600HG
MIR6815
RAD9A
RPS6KA2
RPS6KA3
RPS6KA4
RPS6KC1
RUNX3
SLC44A5
TNFAIP3
TP53BP1
TP53I11
TP63
TRAF7
ZFTRAF1
B cell
oCLL
tCLL

# Figure S4

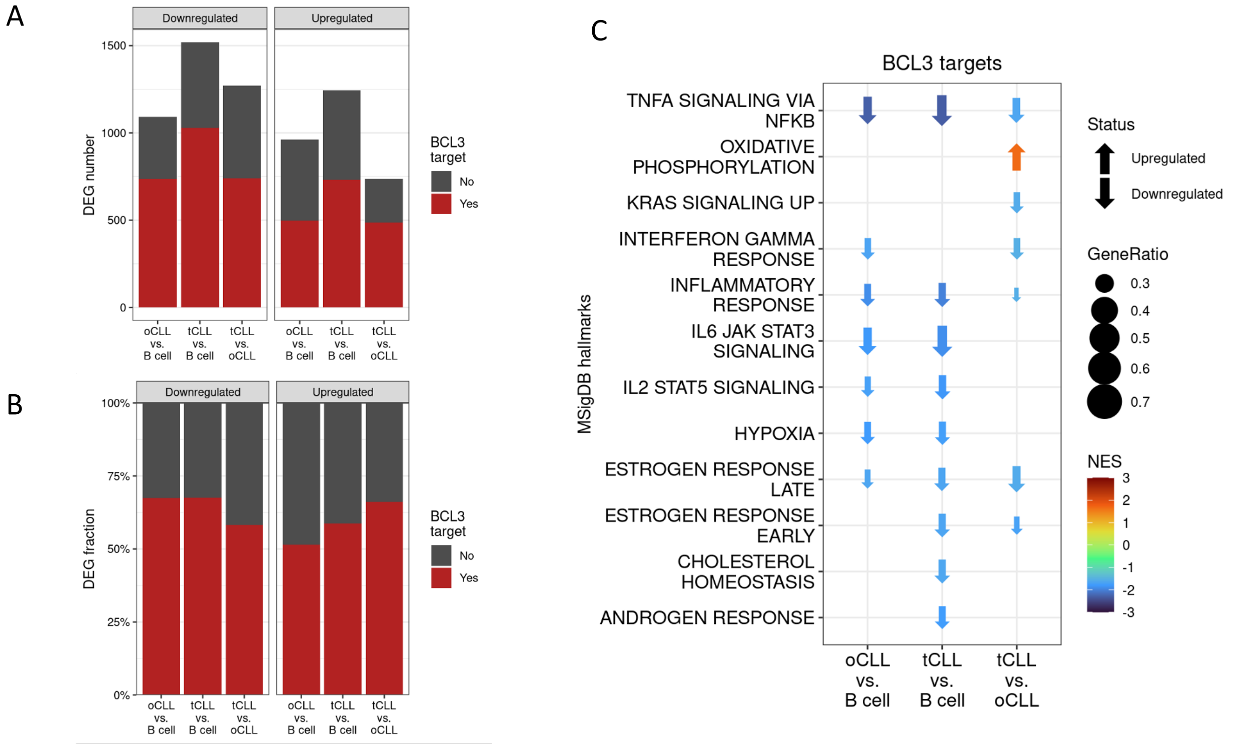


# Figure S5

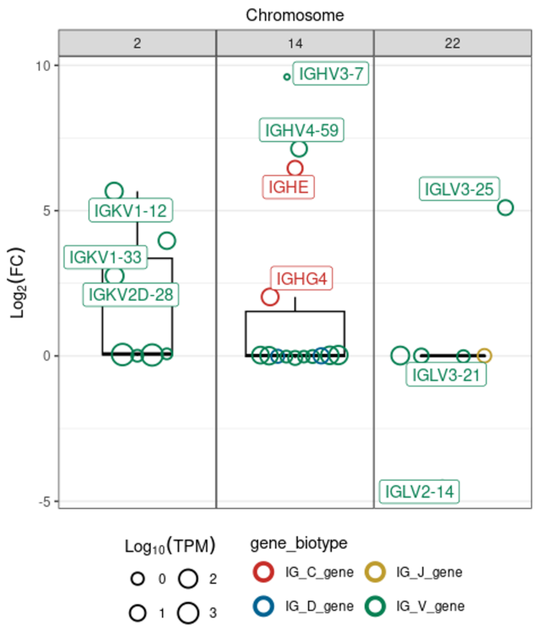

Figure S6

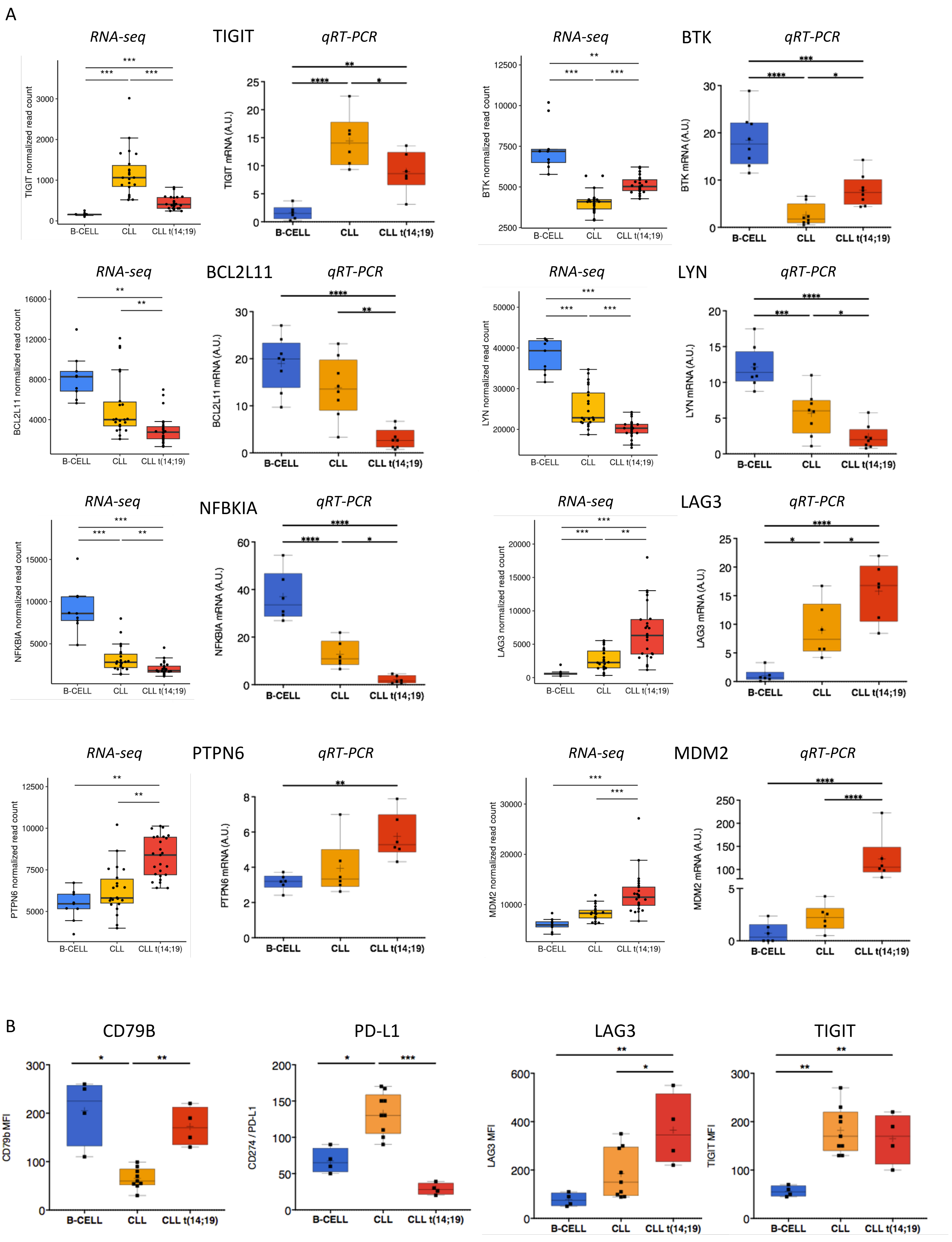
A
TIGIT
BTK
BCL2L11
LYN
NFBKIA
LAG3
PTPN6
MDM2
RNA-seq
qRT-PCR
TIGIT normalized read count
TIGIT mRNA (A.U.)
BTK normalized read count
BTK mRNA (A.U.)
BCL2L11 normalized read count
BCL2L11 mRNA (A.U.)
LYN normalized read count
LYN mRNA (A.U.)
NFKBIA normalized read count
NFKBIA mRNA (A.U.)
LAG3 normalized read count
LAG3 mRNA (A.U.)
PTPN6 normalized read count
PTPN6 mRNA (A.U.)
MDM2 normalized read count
MDM2 mRNA (A.U.)
B-CELL
CLL
CLL t(14;19)
B
CD79B
PD-L1
LAG3
TIGIT
CD79b MFI
CD274 / PD-L1
LAG3 MFI
TIGIT MFI

Figure S7

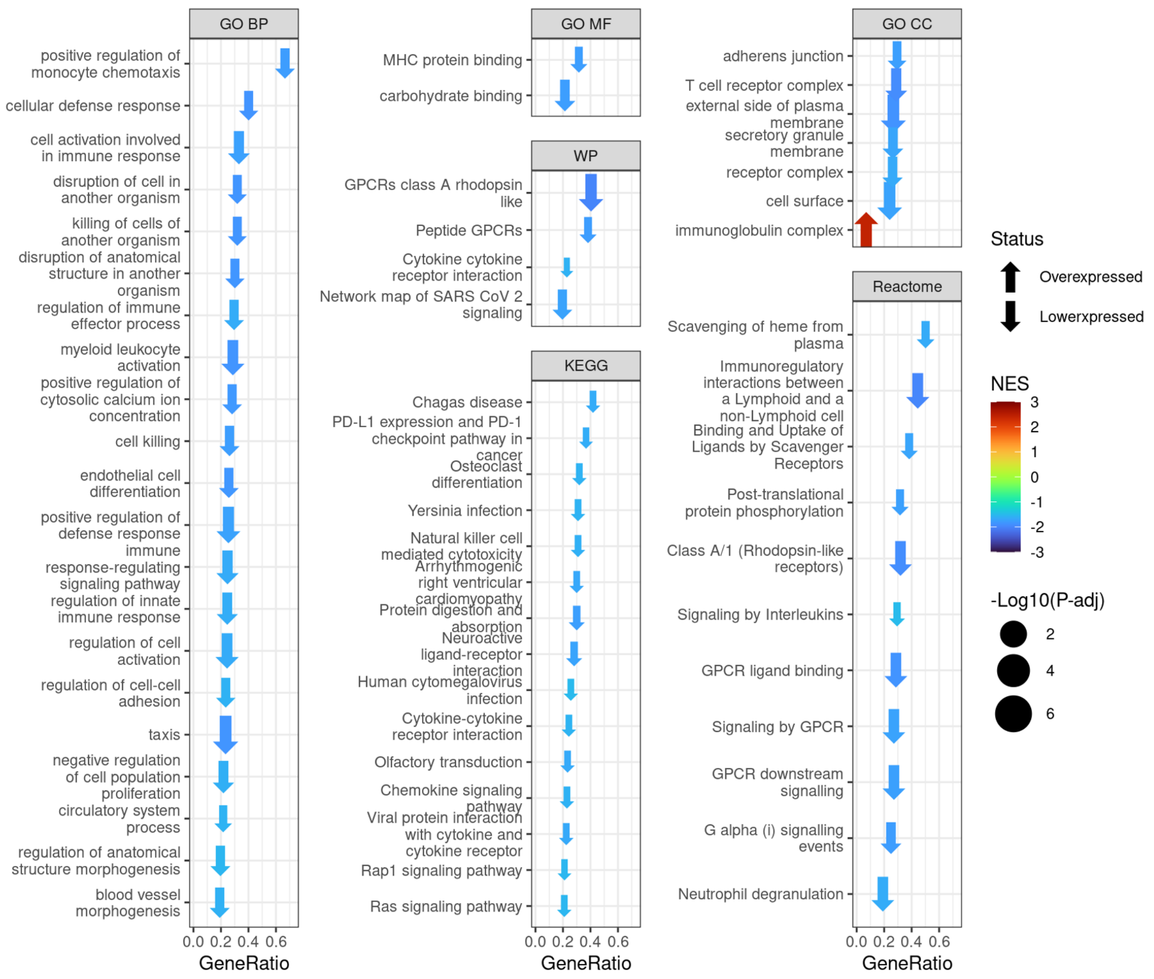


Figure S8

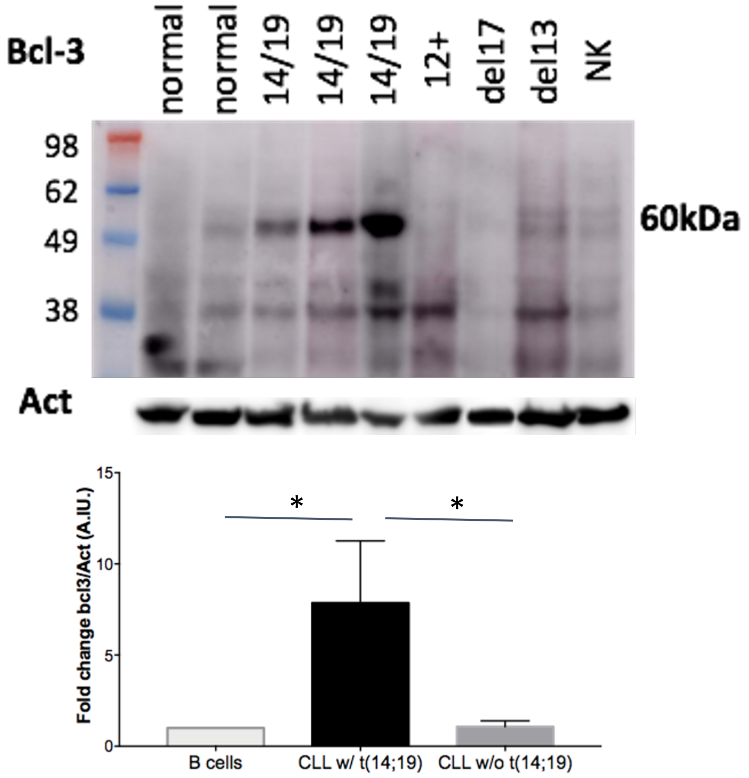

## Supplementary Figure S9

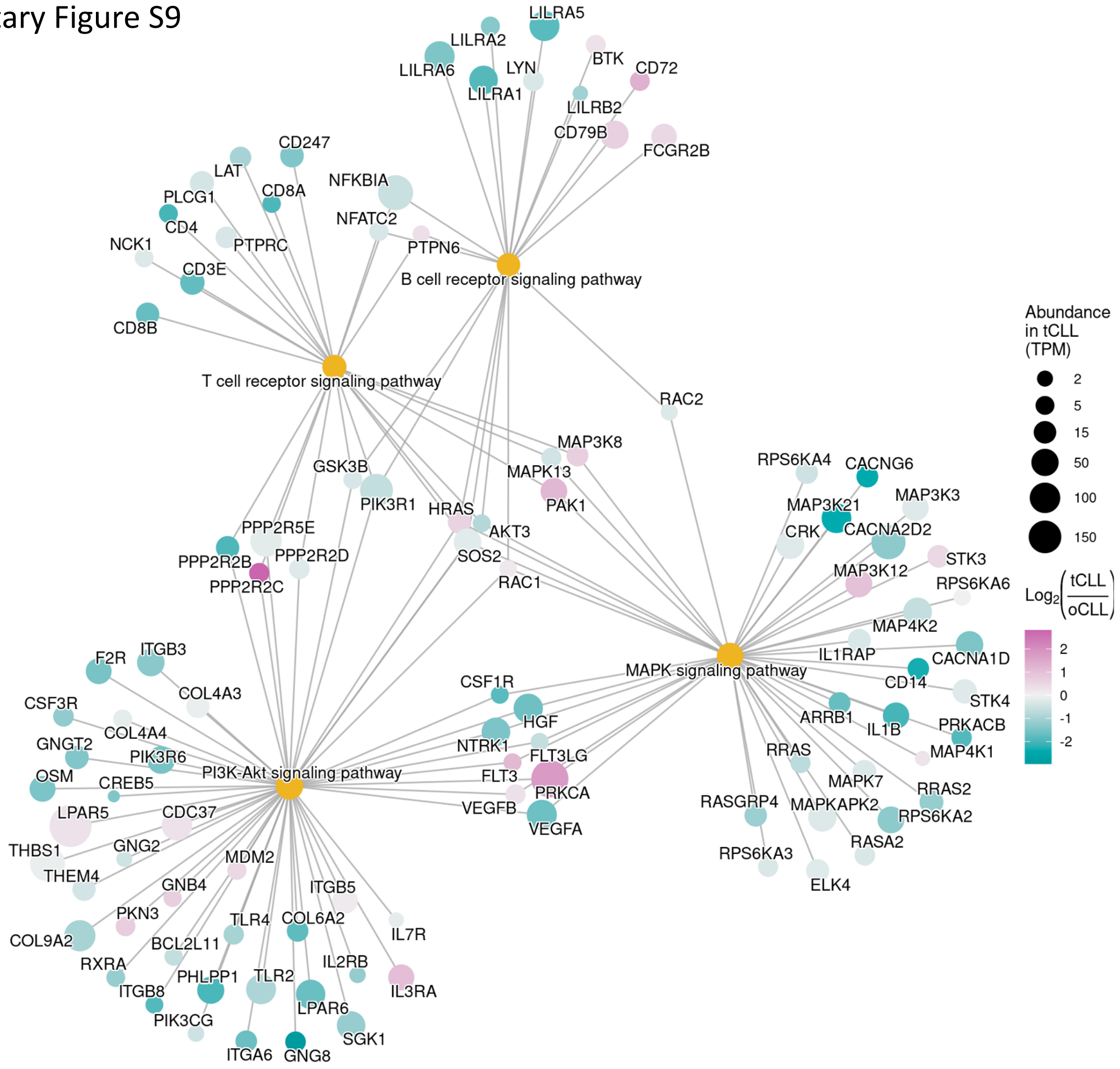


## Supplementary Figure S10

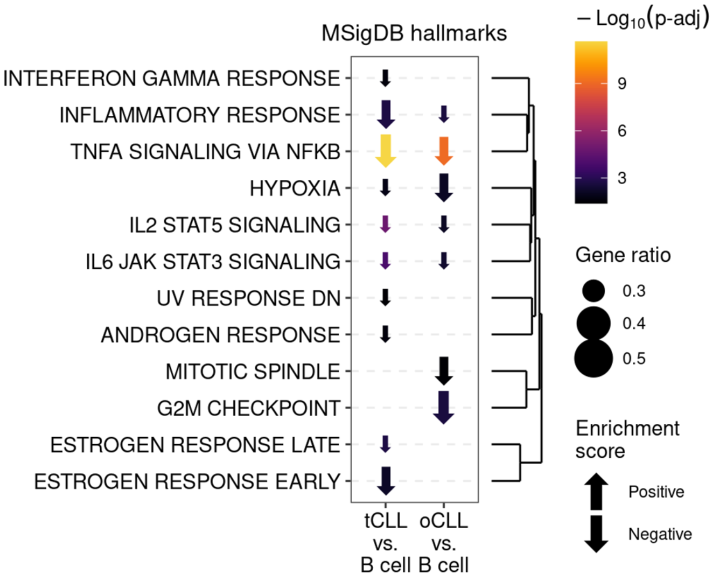


## Supplementary Figure S11

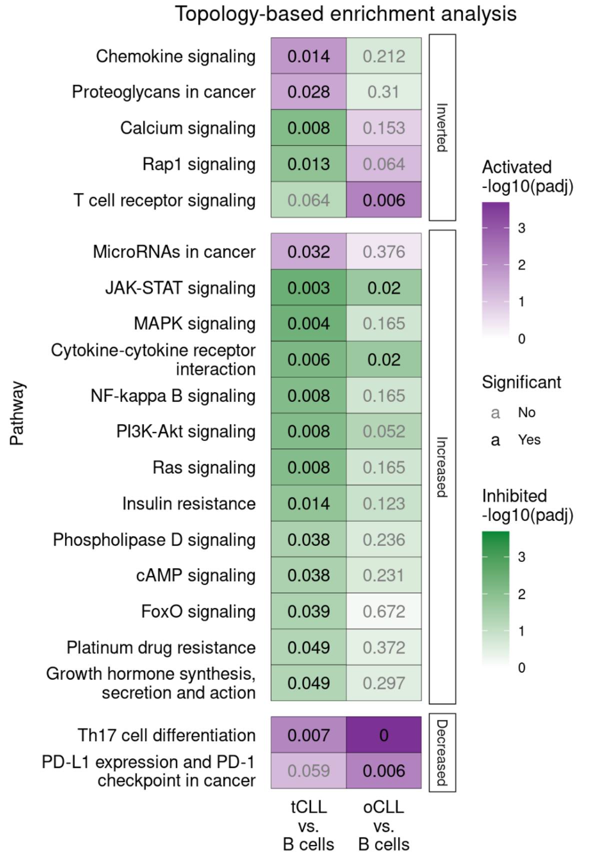

Supplement: Supplementary file 2 — Supplementary Figures [file 41375_2025_2755_MOESM2_ESM.pdf]
